# Subsurface hydrogen as a hidden driver of copper surface reconstruction in CO_2_ electroreduction

**DOI:** 10.1093/nsr/nwag128

**Published:** 2026-03-06

**Authors:** Siwang Zhang, Hang-Biao Lv, Zhong-Zhang Shi, Ruoxuan Wang, Shisheng Zheng, Jian-Feng Li

**Affiliations:** College of Energy, State Key Laboratory of Physical Chemistry of Solid Surfaces, iChEM, College of Chemistry and Chemical Engineering, College of Materials, Institute of Artificial Intelligence, School of Life Sciences, Xiamen University, Xiamen 361000, China; College of Energy, State Key Laboratory of Physical Chemistry of Solid Surfaces, iChEM, College of Chemistry and Chemical Engineering, College of Materials, Institute of Artificial Intelligence, School of Life Sciences, Xiamen University, Xiamen 361000, China; College of Energy, State Key Laboratory of Physical Chemistry of Solid Surfaces, iChEM, College of Chemistry and Chemical Engineering, College of Materials, Institute of Artificial Intelligence, School of Life Sciences, Xiamen University, Xiamen 361000, China; College of Energy, State Key Laboratory of Physical Chemistry of Solid Surfaces, iChEM, College of Chemistry and Chemical Engineering, College of Materials, Institute of Artificial Intelligence, School of Life Sciences, Xiamen University, Xiamen 361000, China; College of Energy, State Key Laboratory of Physical Chemistry of Solid Surfaces, iChEM, College of Chemistry and Chemical Engineering, College of Materials, Institute of Artificial Intelligence, School of Life Sciences, Xiamen University, Xiamen 361000, China; College of Energy, State Key Laboratory of Physical Chemistry of Solid Surfaces, iChEM, College of Chemistry and Chemical Engineering, College of Materials, Institute of Artificial Intelligence, School of Life Sciences, Xiamen University, Xiamen 361000, China; Innovation Laboratory for Sciences and Technologies of Energy Materials of Fujian Province (IKKEM), Xiamen 361000, China

**Keywords:** subsurface H, Cu surface reconstruction, CO_2_ reduction, machine-learning interatomic potentials, grand canonical Monte Carlo

## Abstract

Copper (Cu) undergoes significant surface reconstruction during CO_2_ electroreduction, which is strongly modulated and accelerated by reaction intermediates, yet the atomic-scale mechanism remains far behind the experimental observations. By integrating machine-learning interatomic potentials with large-scale grand canonical Monte Carlo simulations, we systematically investigated *CO- and *H-induced surface roughening across various Cu facets. Our simulations demonstrate that high surface *H (*H_sur_) coverage facilitates subsurface hydrogen (*H_sub_) incorporation on (100)-dominated Cu facets under typical working conditions (−1 V vs. reversible hydrogen electrode), while such penetration is negligible on (111)-like surfaces. This facet dependence is primarily attributed to a *H-induced hexagonal surface reconstruction observed on (100)-dominated facets, a process driven primarily by geometric rather than electronic effects. Specifically, high *H coverage triggers a partial transition of Cu atoms from ideal 4-fold hollow sites to more closely packed 3-fold arrangements. The local densification expands the spacing at the remaining 4-fold sites, thereby reducing the energy barrier for *H_sub_ migration into the subsurface. Further analysis reveals that *H_sub_ alone is sufficient to induce Cu adatom formation, even in the absence of nearby *CO, uncovering a revised structural evolution paradigm for Cu surface roughening. We propose an alloying strategy using low hydrogen affinity metals (Zn, Al, Ga) to effectively suppress *H_sur_ incorporation, offering a promising pathway for designing Cu-based catalysts with long-term stability.

## Teaser

This work reveals how hydrogen atoms burrow into subsurface of the copper catalysts and contribute to the surface roughening.

## INTRODUCTION

The electrochemical carbon dioxide reduction reaction (CO₂RR) is widely regarded as a promising approach toward realizing a carbon-neutral cycle [1–4]. Among various catalysts, copper (Cu) has garnered significant attention due to its unique ability to convert CO₂ into multi-carbon (C_2__+_) products [5]. However, Cu often undergoes pronounced structural reconstruction under reaction conditions, which severely compromises its long-term catalytic stability and remains a critical barrier to practical application [6–9].

Such structural reconstruction of Cu typically initiates with the Cu–Cu bond weakening, followed by adatom formation or dissolution processes [10]. These events are strongly modulated and accelerated by the reaction intermediates on the surface. Among these, *CO, serving as the key intermediate for C–C coupling pathways [11,12], is one of the most crucial species. Under relatively positive potentials [e.g. around −0.2 V vs. a reversible hydrogen electrode (RHE)], locally enriched *CO has been suggested to promote Cu surface restructuring [13,14]. Nevertheless, at more negative potentials typically employed in CO_2_RR (e.g. around −1 V vs. RHE), the steady-state *CO coverage is generally limited to 0.1–0.3 monolayers (ML) [15–17]. This sparse *CO coverage may render it insufficient on its own to induce extensive Cu reconstruction. Meanwhile, at typical negative potentials, the electrolysis of interfacial water becomes inevitable, leading to the generation of a large quantity of hydrogen (*H), which consequently becomes one of the most dominant adsorbed species on the Cu surface [5,18,19]. Surface-adsorbed *H (*H_sur_) alone can moderately weaken Cu–Cu metallic bonding, yet this effect is generally insufficient to trigger significant Cu atom detachment [20,21]. When *H_sur_ is located in proximity to *CO, their cooperative interaction can synergistically weaken the bonding between Cu atoms, thereby facilitating the upward displacement of surface Cu [20]. However, due to the inherently limited *CO coverage and its dynamic turnover under operating conditions, such *CO and *H_sur_ co-adsorption effects are likely to be localized and transient in nature. This consideration calls for closer examination of whether *H alone may contribute to surface destabilization through alternative mechanisms.

Owing to its extremely small size and high mobility, hydrogen is not only capable of adsorbing onto metal surfaces but also can penetrate into subsurface sites, thereby impacting structural stability from beneath the surface. In other transition metal systems such as Pd, Pt and Ti, a broad range of theoretical and experimental studies have demonstrated that *H_sur_ can intercalate into subsurface layers under electrochemical conditions, exerting a profound influence on surface structure and catalytic performance [22–31]. These findings raise the possibility that a similar subsurface *H (*H_sub_) incorporation process may also occur on Cu surfaces. Some experimental investigations have also hinted at the possible presence of *H_sub_ in Cu under electrochemical conditions [32,33]. However, given that the lattice spacing and interstitial volume of Cu are significantly smaller than those of Pd, Pt and Ti, the atomic-scale mechanism by which *H_sur_ penetrates into the Cu subsurface remains unclear. Moreover, the specific impact of *H_sub_ on the structural evolution and catalytic performance of Cu has yet to be systematically elucidated.

Experimentally, direct detection of *H_sub_ remains highly challenging due to its buried location and inherently low scattering cross-section. Conventional surface-sensitive techniques, such as X-ray photoelectron spectroscopy (XPS) and X-ray absorption spectroscopy (XAS), generally lack the spatial and chemical resolution necessary to unambiguously differentiate between *H_sur_ and *H_sub_, particularly under dynamic electrochemical conditions [28]. As a result, current experimental approaches often rely on indirect inference: for instance, by comparing *in situ* XPS or XAS spectra with simulated spectra derived from density functional theory (DFT)-calculated atomic models containing *H_sub_. This strategy, while insightful, is inherently qualitative and limited in its conclusiveness [23,32]. Accordingly, evidence for *H_sub_ in Cu under CO_2_RR conditions remains largely indirect and speculative [32]. From the theoretical perspective, although DFT simulation can directly provide atomic-scale insights, it is often constrained by limited system sizes and timescales, making it difficult to capture *H behavior under high-coverage conditions or to resolve rare events such as subsurface penetration. These limitations underscore the need for a more scalable and dynamic simulation framework capable of resolving the atomistic mechanisms of *H_sub_ formation and assessing its influence on catalyst stability under realistic CO_2_RR conditions.

In this study, we combined machine-learning interatomic potentials (MLIPs) with large-scale grand canonical Monte Carlo (GCMC) simulations to systematically investigate *CO- and *H-induced surface roughening on various Cu facets, including Cu (111), Cu (100), Cu (211) and Cu (711). Leveraging the high efficiency of machine-learning-based force fields, we simulated the dynamic evolution of adsorbates on large surface areas under −1 V vs. RHE. Our results reveal that (100)-like facets exhibit significant *H penetration into the subsurface, whereas such behavior is negligible on (111)-like facets. This disparity is primarily attributed to a surface reconstruction observed on (100)-like surfaces at high *H coverage. Specifically, a subset of Cu atoms transitions from ideal 4-fold hollow sites to more densely packed 3-fold geometries. This localized compression leads to increased spacing at the residual 4-fold sites, effectively reducing the energy barrier for *H_sur_ diffusion into the subsurface. The *H_sub_ alone is capable of inducing Cu adatom formation, even in the absence of nearby *CO. Additionally, we explored how the *H_sub_ influences C_2+_ product selectivity. Finally, we propose that regulating subsurface *H incorporation through alloying may offer a viable strategy to enhance the structural stability and long-term durability of Cu-based CO_2_RR catalysts.

## RESULTS AND DISCUSSION

### The overall workflow

**Figure 1.**
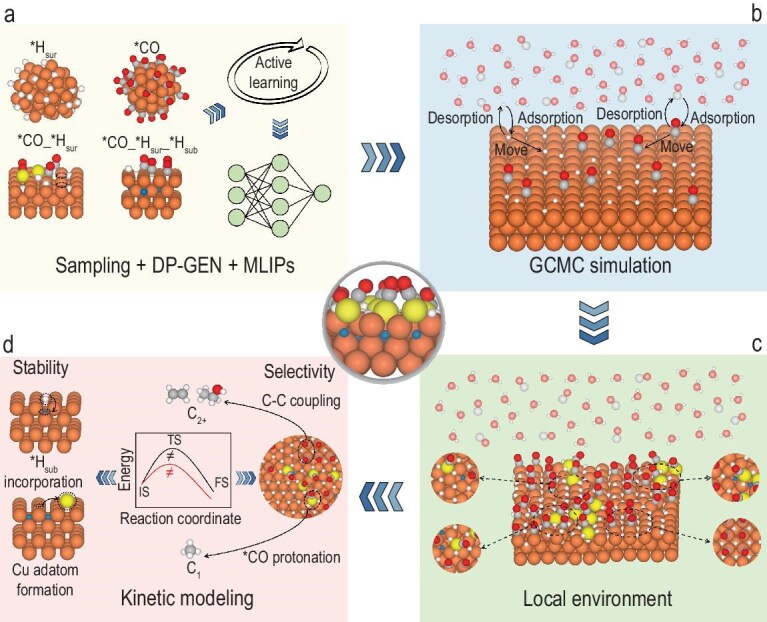
The computational workflow. (a) Development of a high-fidelity MLIPs for the Cu–C–O–H system by an active learning approach via Deep Potential GENerator (DP-GEN). (b) Successive stages in GCMC simulation, with each insertion, deletion and movement action followed by structural relaxation using the MLIPs. (c) Extraction and statistical analysis of the local chemical environments from the GCMC trajectory. (d) Comprehensive evaluation of the system’s dynamics, catalytic activity and stability by kinetic modeling. Brown surfaces are Cu, yellow is adatom Cu, gray is C, red is O, white is surface H, blue is subsurface H. IS: initial state, TS: transition state, FS: final state.

To accurately study the coverage effects of surface adsorbates, it is essential to perform extensive sampling of co-adsorption configurations on sufficiently large surface models, thereby minimizing configurational bias and ensuring statistical reliability [34]. However, such large-scale sampling is computationally prohibitive when relying solely on DFT due to the combinatorial explosion of possible configurations. Recently, MLIPs have emerged as powerful tools for accelerating atomic simulations [35]. By retaining near-DFT accuracy while dramatically improving computational efficiency, MLIPs enable feasible exploration of complex configuration spaces, particularly under high coverage conditions [36–38]. Thus, we initially developed a high-accuracy MLIP covering the Cu–C–O–H chemical space to enable efficient sampling and thermodynamic analysis of *CO and *H co-adsorption on Cu surfaces (Fig. 1a). Subsequently, an MLIP-driven GCMC simulation was employed to model the time-evolved behavior of *CO and *H species under an applied potential of −1 V vs. RHE (Fig. 1b), enabling access to the most probable adsorbate distribution patterns under realistic operating conditions. The GCMC results were then analyzed to assess the overall structural evolution, as well as to statistically characterize local atomic-scale configurations (Fig. 1c). These findings serve as the foundation for subsequent kinetics calculations, allowing evaluation of the surface stability and its impact on the catalytic performance (Fig. 1d). Detailed methodologies for each step are provided in the Methods section and the Supplementary data.

### Cu–C–O–H MLIPs

To construct a reliable potential capable of capturing Cu surface reconstruction, it was necessary to consider a broad diversity of local atomic environments. Therefore, we performed *ab initio* molecular dynamics (AIMD) simulations starting from a set of representative structures, including various Cu surfaces, Cu clusters, adatom-decorated Cu surfaces, *CO, *H_sur_ and *H_sub_ co-adsorption configurations (Figs S1 and S2). The initial Cu–C–O–H dataset consists of 58 740 structures with corresponding DFT-labeled energies and forces. An iterative active learning workflow was applied to further expand the configurational space and improve the robustness of the MLIPs (Fig. S3). For structures where the ML model exhibited insufficient accuracy, self-consistent DFT calculations were performed to re-label these configurations, which were then reintroduced into the dataset. After several iterations, a total of 98 933 unique structures were labeled and incorporated into the final dataset. The resulting MLIPs, trained on the completed dataset, achieve an energy prediction accuracy of 5.42 meV/atom and a force accuracy of 93.5 meV/Å. The reliability and accuracy of the Cu–C–O–H MLIPs were validated in Figs S4–S7, including model uncertainty (*σ*) across four parallel models, the training results of MLIPs, fidelity in reproducing DFT energies and forces, and agreement of radial distribution functions between AIMD and deep potential molecular dynamics (DPMD) simulations.

### Mechanism of *H_sur_ incorporation into Cu subsurface

Leveraging the high computational efficiency of MLIPs, we performed GCMC simulations on four representative Cu surfaces: Cu (111), Cu (100), Cu (211) and Cu (711), each with a surface area of 815, 941, 768 and 840 Å^2^, respectively. The Cu (211) surface consists of (111) terraces and (100) steps, while the Cu (711) surface features (100) terraces and (111) steps, serving as representative high-index facets. GCMC inherently follows the principles of statistical mechanics and circumvents the combinatorial explosion associated with exhaustively enumerating possible equilibrium configurations, making it particularly suitable for modeling coverage-dependent phenomena [34,39].

Simulations were carried out at −1 V vs. RHE, which represents a typical operating potential for Cu-based CO_2_ reduction catalysts. The electrochemical environment is encoded through the chemical potentials of H and CO, which govern their adsorption, desorption and lateral diffusion on the surface (Fig. 1b). Importantly, no explicit constraints or biases were introduced to enforce *H_sur_ incorporation into the subsurface. Instead, we allowed the system to evolve freely to assess whether *H_sur_ would spontaneously migrate into subsurface sites as its surface coverage increases. More technical details can be found in the Methods section and the Supplementary data.

**Figure 2.**
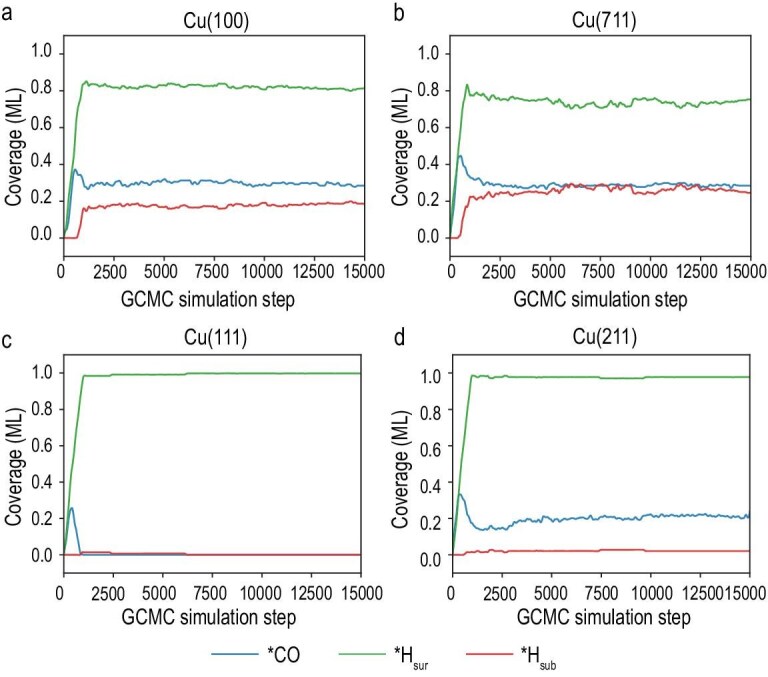
Evolution of surface species during GCMC simulation. Coverage of *CO, *H_sur_ and *H_sub_ as a function of GCMC steps on (a) Cu (100), (b) Cu (711), (c) Cu (111) and (d) Cu (211) facets.

GCMC simulations were performed for 15 000 steps on each of the four Cu surfaces, yielding well-converged results (Fig. 2) with the final surface structure presented in Figs S8–S11. On the Cu (100) surface (Fig. 2a), the *CO surface coverage reaches approximately 0.29 ML, consistent with previous simulation and experiment studies [15–17,20]. In addition, *H_sub_ incorporation is observed at a coverage of 0.18 ML. For Cu (711), which features (100) terraces, similar behavior was found: the *CO coverage is also 0.29 ML. However, the *H_sub_ coverage increased to 0.26 ML, suggesting that the presence of step sites may facilitate *H_sur_ incorporation into the subsurface region (Fig. 2b). Interestingly, on the Cu (111) surface, neither *CO adsorption nor *H_sub_ penetration is observed (Fig. 2c), exhibiting a relatively stable state. This result also aligns with the generally observed low C_2+_ product selectivity on Cu (111), which tends to favor H_2_ and C_1_ products instead. On the Cu (211) surface, the *CO coverage was approximately 0.21 ML, with adsorption sites predominantly located at the step edges (Fig. 2d, Fig. S11). This observation supports the idea that step sites enhance *CO binding, and that Cu (211) is more likely than Cu (111) to promote C_2_ product formation, in line with experimental findings [40]. However, *H_sub_ incorporation on Cu (211) remains negligible, with a coverage of only 0.02 ML. Together, these results suggest that the presence of (100)-like terraces plays a critical role in enabling *H_sub_ incorporation. To confirm the sufficiency of the GCMC simulation, we extended the simulation by an additional 5000 steps. The coverages of *CO, *H_sur_ and *H_sub_, as well as the coordination environments of surface atoms, showed no significant changes at the four facets (Figs S12 and S13). Furthermore, two additional independent trajectories on the representative (100) facet yielded statistically consistent results (Figs S14 and S15). In addition, multiple uniformly subsampled sets and a low-energy subset were also analyzed to further verify the robustness of the GCMC sampling (Figs S16 and S17).

**Figure 3.**
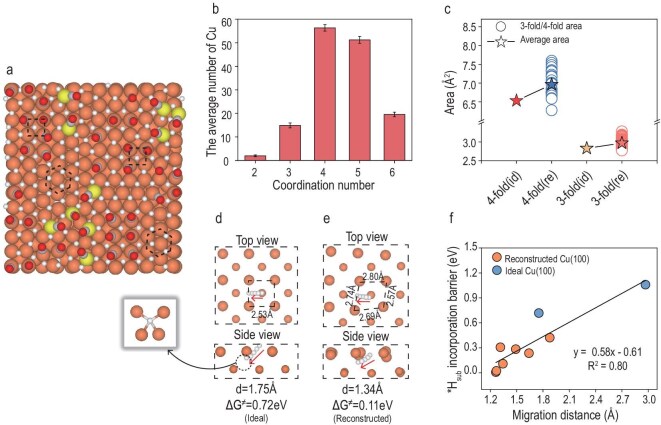
Simulated structural evolution and hydrogen migration behavior on the Cu (100) surface. (a) Atomic configuration of the Cu (100) surface after 15 000 GCMC steps. (b) Distribution of coordination numbers for surface Cu atoms, sampled via 50 representative structures from the last 1000 steps of the GCMC simulation (selected one every 20 steps). (c) Statistical distribution of the surface area for local coordination environments. The blue and orange data points represent the area distributions of the quadrilaterals formed by 4-fold sites and the triangles formed by 3-fold sites, respectively. The stars represent the average areas of the quadrilaterals and triangles formed by the 4-fold and 3-fold sites on both the ideal unreconstructed (id) and reconstructed (re) surfaces. (d) Migration pathway and energy barrier for *H_sur_ incorporation into a subsurface tetrahedral site on the unreconstructed surface. (e) Energy barrier profile for *H_sub_ incorporation within a local reconstructed environment. (f) Correlation between the hydrogen migration barrier and the migration distance into the subsurface. The brown data points represent hydrogen migration in the reconstructed surface environment, whereas the blue data points correspond to migration on the ideal unreconstructed Cu (100) surface (to tetrahedral and octahedral subsurface sites, respectively). Brown surfaces are Cu, small brown ones are subsurface Cu, yellow is adatom Cu, gray is C, red is O, white is H.

To gain deeper insight into the *H-induced reconstruction behavior, we focused our analysis on the Cu (100) surface. After 15 000 steps of GCMC simulation under −1 V vs. RHE, the final morphology of the Cu (100) surface is shown in Fig. 3a. Notably, the surface no longer retains the ideal 4-fold hollow site arrangement but instead exhibits partial transformation into 3-fold hollow sites, forming a mixed structure reminiscent of Cu (111). This local reconstruction emerges progressively with increasing *H coverage, suggesting that *H is the primary driving force behind the structural transition (Fig. S18). To further validate this hypothesis, a comparative GCMC simulation was performed at −0.2 V vs. RHE. Under this condition, the surface is predominantly covered by *CO (0.70 ML) (Fig. S19). The surface maintains the typical 4-fold configuration, confirming that substantial hydrogen adsorption is necessary to trigger this rearrangement of Cu (100).

A statistical analysis of 50 representative structures from the last 1000 steps of the GCMC simulation, sampling one configuration every 20 steps, reveals that approximately 49% of surface Cu atoms had transformed into five- or six-coordinated states, indicating a significant degree of atomic rearrangement towards 3-fold hollow sites (Fig. 3b). Considering the lattice was fixed during GCMC, we further tested whether such reconstruction occurs under more realistic conditions. An ∼2.5 nm Cu nanocluster was constructed and covered with high *H_sur_ coverage for subsequent DPMD simulation. The (100) facets of the cluster underwent similar reconstruction, confirming the robustness of the structural transition (Fig. S20). This phenomenon is supported by experimental observations from Zhu *et al*., who reported that Cu (100) surfaces exhibit (111)-like features after prolonged CO_2_RR operation [41]. Similar reconstructions have also been reported on Pd (100) in the CO_2_RR conditions [22]. This phenomenon bears geometric resemblance to the classical ‘hexagonal reconstruction’ observed on Au (100), Pt (100) and Ir (100) surfaces under thermochemical conditions [42–44]. However, it should be noted that the structures identified in this work are local, disordered and dynamically fluctuating under electrochemical conditions. We therefore refer to them as ‘*H-induced hexagonal reconstructions’ to emphasize the geometric tendency rather than strict crystallographic symmetry. This suggests a converging structural evolution pathway across different chemical environments. Namely, surface atoms tend to reorganize into more closely packed, (111)-like configurations that are inherently more thermodynamically stable than the (100) arrangement. Thus, whether driven by thermal energy or *H-induced surface stress, the reconstruction likely reflects a shared energetic preference for local (111)-type ordering in face-centered cubic (FCC) metals.

In conjunction with the *H-induced hexagonal reconstruction, we further analyzed the kinetics of *H_sur_ migration into the Cu subsurface. We first focused on an ideal unreconstructed Cu (100) 3 × 3 supercell to build a baseline. Two representative diffusion pathways were considered: *H_sur_ migrating into either an octahedral or a tetrahedral subsurface site. Nudged elastic band (NEB) calculations yielded energy barriers of 1.06 and 0.72 eV, respectively, indicating that *H_sub_ incorporation is kinetically unfavorable on a pristine Cu (100) surface (Fig. S21b, Fig. 3d). However, after *H-induced hexagonal reconstruction, the local atomic arrangement is significantly altered. Partial transformation of Cu atoms into more densely packed 3-fold domains leads to lateral expansion in the remaining 4-fold regions, resulting in increased Cu–Cu distances and enlarged local surface area. Statistical analysis (Fig. 3c) shows that the average area of the transformed 3-fold sites is 2.97 Å^2^, while the preserved 4-fold sites expand to an average area of 6.96 Å^2^, significantly larger than the ideal unreconstructed 4-fold site area of 6.53 Å^2^. Representatively, in locally preserved 4-fold hollow regions, the Cu–Cu bond length exceeds the ideal value of 2.53 Å, reaching up to 2.80 Å (Fig. 3e). This geometric dilation allows *H_sur_ to sink deeper into the surface, thereby reducing the barrier for subsurface migration. Specifically, the energy barrier for *H_sur_ migrating into a subsurface tetrahedral site in the reconstructed region was found to be only 0.11 eV, suggesting a nearly barrierless process and highlighting the high feasibility of subsurface incorporation. Further analysis across different 4-fold sites reveals that the migration barrier strongly correlates with the distance between the initial surface site and the final subsurface site—shorter distances correspond to lower transition-state energies (Fig. 3f, Fig. S23). This geometric–kinetic relationship underscores how local geometric relaxation from hexagonal reconstruction facilitates *H_sub_ incorporation and provides a mechanistic basis for this behavior. Interestingly, the reconstructed 3-fold surface environments also exhibit lower migration energy barriers for *H_sub_ insertion than those on an ideal Cu (111) surface due to the elongated Cu–Cu bonds (Fig. S24), further highlighting the importance of structural deformation in governing migration behavior. We further employed grand canonical DFT (GC-DFT) to investigate the relationship between the *H_sub_ incorporation energy barrier and the applied potential. The results suggest that the applied potential has no significant influence on the *H_sub_ incorporation barrier (Fig. S25).

Similar *H-induced hexagonal reconstruction was also observed on the Cu (711) surface, indicating that this behavior is not exclusive to the ideal Cu (100) facet but exhibits broader geometric adaptability (Figs S9 and S26a). This provides a rational explanation for the significant *H_sub_ incorporation observed on Cu (711). In contrast, Cu (111) and Cu (211) surfaces are characterized by compact atomic arrangements dominated by 3-fold hollow sites, which are inherently more stable and lack the geometric flexibility needed for reconstruction under high *H coverage (Fig. S26b and c). Our calculations show that the energy barriers for *H_sur_ insertion into these two surfaces are comparable to those of ideal Cu (100), consistent with their limited ability to host *H_sub_ (Fig. S21). Notably, although Cu (211) is primarily composed of 3-fold coordinated regions, it features a small number of 4-fold step-edge sites that may locally facilitate *H_sub_ incorporation, explaining the small fraction of *H_sub_ in GCMC simulations.

### *H_sub_ accelerates the Cu adatom formation

After elucidating the atomic-scale mechanism of *H_sub_ penetration, we further investigated how *H_sub_ affects the stability of surface Cu atoms on the Cu (100) system. We categorized surface Cu atoms of the 50 representative structures based on their local adsorption environments—specifically, whether *CO is adsorbed and the numbers and types of *H coordination (surface vs. subsurface).

**Figure 4.**
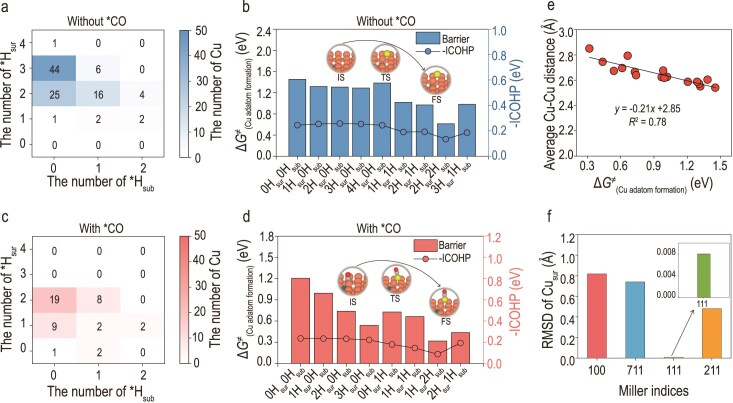
Influence of the local atomic environment on Cu adatom formation behavior. (a and c) Statistical distributions of surface Cu environments on the Cu (100) facet obtained from GCMC simulations in the absence and presence of *CO adsorption, respectively. (b and d) Corresponding Cu adatom formation energy barriers [Δ*G*^‡^_(Cu Adatom Formation)_, bars] and ICOHP values (lines) of Cu–Cu bonds. Detailed calculation configurations are presented in Figs S27 and S29. (e) Correlation between the mean Cu–Cu bond distance and the Cu adatom formation energy barrier. (f) RMSD distributions of surface Cu atoms on the Cu (100), Cu (111), Cu (211) and Cu (711) facets. Brown surfaces are Cu, yellow is adatom Cu, gray is C, red is O, white is surface H, blue is subsurface H.

For surface Cu atoms without adsorbed *CO, most are coordinated with *H (Fig. 4a). In typical environments where Cu is adjacent to 2 or 3 *H_sur_ atoms, the computed Cu adatom formation barriers are comparable to those without any adsorbates at around 1.45 eV (Fig. 4b, Fig. S27), indicating that *H_sur_ alone has a limited destabilizing effect on Cu, consistent with previous studies [20]. However, when at least one *H_sub_ atom is present near the Cu site (e.g. 2*H_sur_ + 1*H_sub_), the adatom formation barrier significantly decreases to 0.97 eV. In the case of two *H_sub_ atoms (2*H_sur_ + 2*H_sub_), the barrier further drops to 0.61 eV, suggesting that *H_sub_ alone can serve as an effective driving force for Cu atom destabilization under reaction conditions, even in the absence of *CO. We also revealed that *H_sub_ exhibits a greater driving force for the formation of Cu adatoms compared to *H_sur_ across other crystal facets (Fig. S28). In *CO-coordinated Cu atoms, we observed a similar trend: the position of *H_sub_ critically influences the extent of destabilization (Fig. 4c and d, Fig. S29). For example, when Cu is coordinated with one *CO and one *H, the Cu adatom formation barrier is ∼1.0 eV if the *H is *H_sur_, but decreases to ∼0.73 eV if the *H is *H_sub_. Overall, the coexistence of *CO and *H (either *H_sur_ or *H_sub_) lowers the energy barrier for Cu adatom formation compared to the presence of *CO or *H alone (Fig. 4b, Fig. S30), which underscores the synergistic effect between *CO and *H. Given that the Cu (111) facet is also a common substrate for the CO_2_RR, we have validated this conclusion on the Cu (111) facet by manually constructing relevant local environments (Fig. S31), even though *H_sub_ and *CO were not observed in the GCMC simulations.

To further investigate the structure–stability relationship, we evaluated the bonding interactions between the uplift Cu atom and its first neighbors using the integrated crystal orbital Hamilton population (ICOHP). The results show a positive correlation between Cu–Cu bond strength and the adatom formation barrier, confirming that *H promotes Cu destabilization primarily by weakening Cu–Cu metallic bonding. *H_sub_ exhibits a stronger bond-weakening effect compared to *H_sur_ (Fig. 4b and d). Additionally, statistical analysis of average Cu–Cu bond lengths in the local environment of the uplift atom with the Cu adatom formation barrier supports the same trend (Fig. 4e). We also computed the root-mean-square displacement (RMSD) of surface Cu atoms from their initial positions for all four facets (Fig. 4f). Cu (100) and Cu (711), which feature significant *H_sub_ penetration, exhibit larger RMSD values, indicating pronounced surface atom displacement. In contrast, Cu (211), which hosts only surface *H_sur_ and *CO, shows moderate movement, while Cu (111) displays minimal atomic movement. These observations collectively highlight that *H_sub_, although not directly involved in typical catalytic steps, exerts a profound influence on surface stability, acting as a previously overlooked structural driver.

### The impact of *H_sub_ on product selectivity

**Figure 5.**
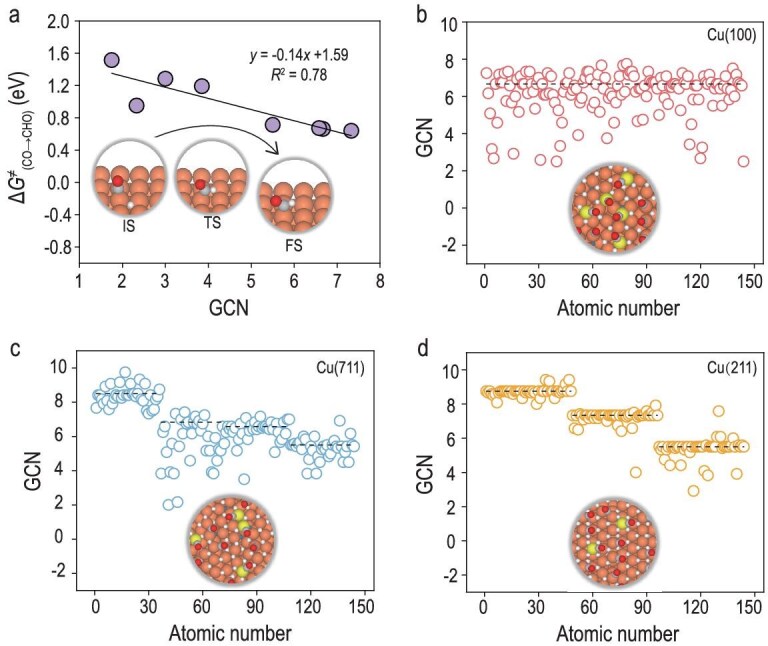
Relationship between the GCN and catalytic properties. (a) Energy barrier for the hydrogenation of *CO to *CHO [Δ*G*^‡^_(CO→CHO__)_] as a function of GCN. (b–d) GCN distribution of surface atoms from simulations of the (b) Cu (100), (c) Cu (711) and (d) Cu (211) facets. The dashed lines represent the theoretical GCN values of the surface Cu atoms in the pristine slab models. Notably, high-index facets contain multiple distinct types of Cu sites, resulting in several values, whereas low-index facets typically exhibit only a single type of site. 6.67 for Cu (100); 8.5, 6.83, 6.58 and 5.5 for Cu (711); and 8.75, 7.33 and 5.5 for Cu (211). Brown surfaces are Cu, yellow is adatom Cu, gray is C, red is O, white is surface H.

Previous studies have shown that C–C coupling barriers are generally lower on Cu sites with reduced generalized coordination numbers (GCNs) [45,46]. Since the formation of C_2+_ products not only requires facile C–C coupling but also suppression of the *CO hydrogenation pathway (leading to C_1_ products), we further calculated the hydrogenation barriers of *CO by *H_sur_ on Cu atoms with different GCNs (Fig. 5a, Fig. S32). The results indicate that lower-GCN sites also exhibit higher barriers for *CO hydrogenation, making them more favorable for retaining *CO and promoting C–C coupling.

In our GCMC simulations of Cu (100) under −1 V vs. RHE conditions, we observed significant surface copper reconstruction driven by *H_sub_ incorporation. The GCN distribution of surface Cu atoms after simulation reveals a notable emergence of low-GCN sites, deviating from the pristine Cu (100) GCN of 6.67 (Fig. 5b, Fig. 3a). This structural evolution likely contributes to the enhanced C_2+_ selectivity of Cu (100) under reaction conditions. A similar trend is observed on Cu (711), where GCMC sampling leads to an even higher density of low-GCN sites compared to its already undercoordinated pristine structure (Fig. 5c, Fig. S9). In contrast, Cu (211), which only exhibits minor *H_sub_ penetration, shows a relatively small change in its GCN distribution before and after simulation (Fig. 5d, Fig. S11). This highlights the critical role of *H_sub_ in generating low-GCN sites and thus further impacts on their C_2+_ selectivity.

### Alloying strategy to suppress *H_sub_ incorporation

The incorporation of *H_sub_ on Cu (100) can induce local surface reconstruction, which generates low-GCN Cu sites and thus favors C–C coupling towards C_2+_ product. But it also compromises the long-term structural stability of the catalyst. This highlights a typical activity–stability trade-off in electrocatalysis: structural activation enhances reactivity but may accelerate surface Cu atom migration, dissolution and irreversible reconstruction, ultimately leading to catalyst degradation [10]. The *H_sub_ is not the sole contributor to Cu restructuring. Other factors, including interfacial water, local electric fields from cations, and co-adsorbed *CO, also play key roles [10,47]. Furthermore, previous studies have shown that *H_sub_ may diffuse deeper into the metal lattice under prolonged electrochemical conditions, leading to hydrogen embrittlement and metal fragmentation [29]. Therefore, moderately suppressing *H incorporation into the subsurface is critical for enhancing catalyst durability without activity scarcity.

**Figure 6.**
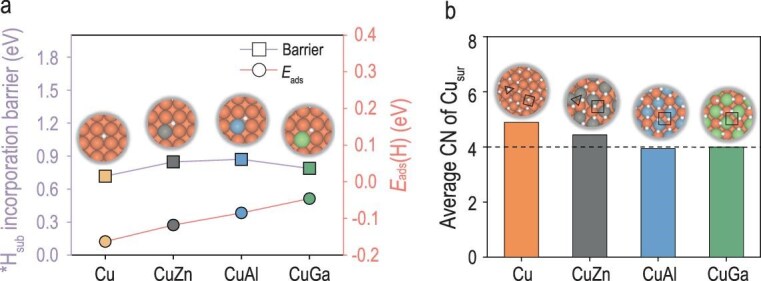
*H_sub_ incorporation barrier and surface atomic coordination in Cu-based alloy systems. (a) Calculated *H_sub_ incorporation barriers (left axis) and *H_sur_ adsorption energies [*E*_ads_(H), right axis] on Cu and Cu–M (M = Zn, Al, Ga) alloy surfaces with one dopant atom. (b) Average coordination numbers of surface atoms obtained from Cu–M alloy models (M = Zn, Al, Ga) constructed using a 6 × 6 supercell with 50% surface doping (Cu:M = 1:1) after 10 ps of AIMD simulations. Brown surfaces are Cu, gray is Zn, light blue is Al, green is Ga, white is surface H.

To this end, we propose an alloying strategy to inhibit *H_sub_ incorporation as a means of balancing activity and stability. Specifically, introducing metal dopants with low hydrogen affinity can reduce hydrogen adsorption on the Cu surface, thereby decreasing *H_sur_ coverage and hindering its penetration into the subsurface. We selected Zn, Al and Ga as representative low-hydrogen-affinity dopants to evaluate their effects [48]. Model calculations show that introducing a single dopant atom into Cu (100) can make the *H_sur_ adsorption energy more positive, indicating weaker *H_sur_ binding and thus lower surface coverage, potentially freeing up more active Cu sites for *CO coupling (Fig. 6a). Moreover, the energy barrier for *H_sur_ migration to subsurface tetrahedral sites increases notably upon doping, suggesting suppressed *H_sub_ penetration kinetics (Fig. 6a, Fig. S33). Since we identified that the hexagonal surface reconstruction on Cu (100) is a prerequisite for *H_sub_ incorporation, we further used a 6 × 6 supercell with 50% surface doping to mimic realistic alloy environments with 1 ML *H_sur_ and performed AIMD simulations to assess the suppression of this reconstruction. Results show that Cu atoms in CuZn, CuAl and CuGa systems maintain higher coordination environments, and hexagonal restructuring is largely inhibited, thus reducing the likelihood of *H_sur_ entering the subsurface (Fig. 6b, Fig. S34). This inhibition is attributable not only to the weaker interaction between Zn, Al, Ga and *H, but also to their larger atomic radii compared to Cu.

These findings provide a mechanistic rationale for the experimentally observed stability and activity of Cu-based alloys. Compared to pure Cu, CuZn, CuAl and CuGa alloys exhibit enhanced structural robustness under operating conditions, without showing pronounced segregation of the alloying elements [49–52]. Therefore, alloying is not merely an electronic tuning strategy. It also offers a structural pathway to mitigate surface reconstruction and improve catalyst longevity, enabling the simultaneous realization of high activity and long-term stability. It should be noted that the optimal dopant concentration was not systematically explored in this study and remains an open question for future experimental optimization. Additionally, other metals with similar low hydrogen affinity, such as Mg and Sn, represent promising candidates for constructing stable and efficient Cu-based alloy catalysts and warrant further investigation.

Taken together, our findings identify *H_sub_ as a hidden yet decisive driver of Cu surface reconstruction under CO_2_RR conditions. The term ‘hidden’ is multidimensional in nature. Physically, *H resides beneath the surface, evading direct detection by conventional surface-sensitive techniques and typically inferred only through indirect evidence [23,32]. Mechanistically, its influence has remained largely overlooked, with prior attention predominantly focused on surface-bound species such as *CO and *H_sur_. Our simulation results challenge this prevailing view and instead point to the need for a new structural evolution paradigm, which arises from the dynamic coupling between surface and subsurface intermediates. Conceptually, it exemplifies a broader category of elusive factors that quietly yet profoundly shape catalytic behavior. Notably, similar concepts are beginning to gain traction in the hydrogen evolution reaction community as well [23]. Therefore, it may open new frontiers for understanding and controlling catalyst evolution across a broad spectrum of electrochemical transformations.

We note that several aspects of the present work remain open for further investigation. Subsurface hydrogen incorporation is strongly influenced by pH. Obviously, this process is most favorable under acidic conditions due to the higher *H_sur_ coverage, and becomes progressively less favorable and is expected to be suppressed as the pH increases. In addition, variations in pH can introduce other interfacial species. For example, under near-neutral conditions, *OH has also been suggested to contribute to Cu surface reconstruction [53]. A systematic understanding of the pH dependence of *H_sub_ incorporation and its competition with alternative reaction pathways remains an important direction for future studies. Our work primarily focuses on the feasibility of *H_sub_ incorporation at −1 V vs. RHE. Investigating the potential dependence of this process, particularly identifying the critical potential at which *H_sub_ emerges during the transition from positive to negative potentials, represents another direction for future research. In addition, from an experimental perspective, beyond identifying the presence of *H_sub_ through *in situ* XPS and XAS combined with DFT-simulated spectral comparisons [23,32], this behavior may also be probed using energy-loss and UV–visible spectroscopy, high-sensitivity mass-change measurements based on quartz crystal microbalance, and characteristic features in cyclic voltammetry under specific conditions [28]. An integrated experimental strategy that combines these complementary techniques would be highly valuable for improving confidence in identifying *H_sub_ and for deepening mechanistic insights into hydrogen-induced surface evolution in CO_2_RR.

## CONCLUSIONS

In this work, we systematically investigated *CO- and *H-induced surface roughening on various Cu facets, including (100), (111), (711) and (211), by combining MLIPs with large-scale GCMC simulations. Our results reveal that (100)-dominated facets, typically associated with high C_2_ product selectivity, exhibit significant *H penetration into the subsurface, whereas such behavior is negligible on (111)-like facets. This facet-dependent trend is primarily attributed to *H-induced hexagonal surface reconstruction observed on Cu (100) and Cu (711). Specifically, high *H coverage triggers a partial transition of Cu atoms from ideal 4-fold hollow sites to more closely packed 3-fold arrangements. This local densification results in expanded spacing at the remaining 4-fold sites, thereby lowering the energy barrier for *H_sur_ migration into the subsurface. Further analysis shows that *H_sub_ alone is sufficient to induce Cu adatom formation, even without nearby *CO. When *CO is co-adsorbed, *H_sub_ can further promote surface Cu detachment. Additionally, we find that *H-induced restructuring creates low-coordination Cu sites, which may facilitate C–C coupling and enhancing C_2+_ product formation on Cu (100). Finally, we propose that suppressing *H_sub_ incorporation by alloying metals of low hydrogen affinity offers a promising route to mitigate structural degradation while preserving catalytic activity. Together, these findings provide atomic-level insight into the origins of Cu surface reconstruction under CO_2_RR conditions and establish a mechanistic foundation for designing more robust and selective Cu-based catalysts.

## METHODS

### DFT calculations

DFT calculations were performed using the Vienna *Ab initio* Simulation Package (VASP) [54]. The revPBE [55] exchange–correlation functional and the projector augmented-wave (PAW) [56] method were employed. A plane-wave kinetic energy cutoff of 400 eV and a 2 × 2 × 1 Monkhorst–Pack k-point mesh were used for Brillouin zone sampling. A vacuum layer of 15 Å was applied along the surface normal to avoid periodic interactions. Structural optimizations were conducted with convergence thresholds of 0.05 eV/Å for atomic forces and 10⁻⁵ eV for the total energy. The van der Waals interactions were accounted for by Grimme’s DFT-D3 dispersion correction [57]. The energy barriers for Cu adatom formation, *H migration and *CO protonation were determined via the climbing-image NEB (CI-NEB) method [58]. Each transition state was confirmed by the presence of exactly one imaginary frequency in the vibrational spectrum. The crystal orbital Hamilton population (COHP) analysis was carried out with the LOBSTER code to evaluate Cu–Cu bonding strength [59,60]. The detailed description of the DFT calculation model is available in the Supplementary data. In this work, MLIPs are primarily employed in GCMC simulations. Other critical calculations, including hydrogen migration barriers, copper adatom formation barriers and CO protonation barriers, are performed using DFT.

### Techniques for MLIPs

To ensure the comprehensiveness and accuracy of the initial dataset, we employed an active learning algorithm implemented in the DP-GEN package [61]. In each iteration, four machine-learned force field models were trained to collectively explore the potential energy surface. The initial dataset was constructed through short AIMD simulations in the canonical (NVT) ensemble using a Nosé–Hoover thermostat [62,63], where each structure underwent 20 simulation steps. Configuration space sampling was subsequently performed via molecular dynamics (MD) simulations using the LAMMPS package [64] integrated with the DeePMD method. Candidate configurations were screened from MD trajectories based on model deviations evaluated from the maximum force predictions, with a confidence threshold defined between 0.10 and 0.35 eV/Å. The final dataset consisted of 98 933 unique structures, yielding an energy prediction accuracy of 5.42 meV/atom and a force prediction accuracy of 93.5 meV/Å. This accuracy is comparable to that achieved in similar systems reported in recent studies [39,65]. Comprehensive details regarding the techniques for MLIPs are provided in the Supplementary data.

The initial configurations of the dataset were generated using our in-house developed PH-SA [22] sampling method. This approach accurately identifies both surface and bulk adsorption sites, on which adsorbate molecules are subsequently placed to construct the complete adsorption systems. A detailed description of the sampling procedure is provided in the ‘Initial dataset’ subsection within the ‘Techniques for Machine Learning Interatomic Potentials (MLIPs)’ section of the Computational Methods in the Supplementary data.

### GCMC simulation

GCMC simulations [66] were conducted to study the co-adsorption equilibrium of *CO and *H on Cu surfaces within the constant chemical potential (*μ*), volume (*V*) and temperature (*T*) ensemble. The system explores equilibrium configurations through three types of Monte Carlo trials: particle displacement, insertion and deletion. To overcome sampling limitations, structural relaxation driven by MLIPs was introduced after each step [67,68], significantly enhancing phase space exploration while maintaining thermodynamic consistency.

GCMC simulations were performed on four representative Cu surfaces: Cu (100), Cu (111), Cu (211) [featuring (111) terraces and (100) steps] and Cu (711) [featuring (100) terraces and (111) steps]. Each surface was constructed with a 15 Å vacuum layer along the *z*-direction to minimize periodic interactions. The Cu (100) and Cu (111) surfaces were modeled using 3-layer 12 × 12 supercells, while the Cu (211) and Cu (711) surfaces were represented by 3-layer 4 × 12 and 3-layer 3 × 12 supercells, respectively. The surface areas of Cu (111), Cu (100), Cu (211) and Cu (711) are 815, 941, 768 and 840 Å^2^, respectively. Comprehensive details regarding the GCMC simulation methodology are provided in the Supplementary data.

## Supplementary Material

nwag128_Supplemental_File

## Data Availability

The force field training dataset, the trained force field model, and the relevant calculation files for *H_sub_ incorporation barriers, Cu adatom formation barriers, hydrogen adsorption energies and CO protonation are available on Zenodo at https://doi.org/10.5281/zenodo.18375029 [69]. Source data for this paper are also provided therein.
