# Supplementary material for: Subsurface hydrogen as a hidden driver of copper surface reconstruction in CO2 electroreduction
Source: Natl Sci Rev. 2026 Mar 6;13(9):nwag128. doi: 10.1093/nsr/nwag128 (PMC13214988; doi:10.1093/nsr/nwag128)
Supplement: nwag128_Supplemental_File [file nwag128_supplemental_file.pdf]

Subsurface Hydrogen as a Hidden Driver of Copper Surface Reconstruction in CO_2 Electroreduction

Siwang Zhang[1], Hang-Biao Lv[1], Zhong-Zhang Shi[1], Ruoxuan Wang[1], Shisheng Zheng [*1], Jian-Feng Li [*1, 2]

[1] College of Energy, State Key Laboratory of Physical Chemistry of Solid Surfaces, iChEM, College of Chemistry and Chemical Engineering, College of Materials, Institute of Artificial Intelligence, School of Life Sciences, Xiamen University, Xiamen 361000, China

[2] Innovation Laboratory for Sciences and Technologies of Energy Materials of Fujian Province (IKKEM), Xiamen 361000, China

*Corresponding author: zhengss@xmu.edu.cn (S.S.Z.); Li@xmu.edu.cn (J.L.)

Computational Methods

Techniques for Machine Learning Interatomic Potentials (MLIPs)

The Deep Potential GENerator (DP-GEN)[1] was employed to construct the dataset for the adsorption systems of *CO and *H on Cu catalysts. DP-GEN is a learning scheme that operates iteratively. Each iteration of the data generation process consists of three stages: training, exploration, and labeling. The detailed workflow is illustrated as follows.

Initial dataset. The initial dataset was constructed to train a preliminary Deep Potential (DP) model for navigating configuration space exploration and screening candidate structures. A comprehensive set of copper-based models was constructed to systematically probe structure-dependent catalytic behavior, including periodic slabs of Cu(100) and Cu(111) as 4-layer 3×3 supercells; Cu(110) as a 2-layer 2×4 supercell;

Cu(210), Cu(211), Cu(711) as 3-layer 1×3 supercells; Cu(311) as a 3-layer 3×3 supercell; and Cu(810) as a 3-layer 1×2 supercell. Additionally, a Cu_{55} nanoparticle—both in its pristine form and after reconstruction via 5 ps AIMD at 1200 K—was included. To further examine the role of surface defects, adatom-vacancy configurations were modeled using 3-layer 4×4 supercells for Cu(100) and Cu(111), 3-layer 1×4 supercells for Cu(211) and Cu(711), and a 3-layer 1×3 supercell for Cu(810), each incorporating varying numbers of Cu adatoms with corresponding vacancies.

We applied our developed site identification method (PH-SA[2]) to identify surface and subsurface sites on both the original and reconstructed models shown in Figure S1. *H and *CO were then adsorbed onto these sites to form *CO, $*H_{sur}$, and $*H_{sub}$ co-adsorption configurations. Five types of co-adsorption configurations for the different models are illustrated in Figure S2.

For each model, the number of surface atoms m was first determined. The coverage gradient was then categorized according to $CO\_x\_H_{sur}\_y\_H_{sub}\_z$ (where $x + y \leq m$, $z \leq$ 0.3ML). For each coverage level, 200 unique configurations were systematically enumerated. Redundant configurations were eliminated through graph isomorphism detection: each generated configuration was converted into an atomic graph based on atomic radii and connectivity. Each graph underwent three iterations of the Weisfeiler–Lehman (WL) transformation using the WL algorithm[3]. Finally, graph isomorphism was assessed using the isomorphism algorithm in networkx.

The 200 unique configurations identified for each coverage level were converted into node graphs, and pairwise similarity measures between these graphs were computed. The resulting similarity matrix was subjected to principal component analysis (PCA) to perform clustering. From the resulting clusters, ten representative configurations were selected and used in AIMD simulations with 20 steps. The time step is 1 fs. From the resulting trajectories, configurations were extracted every 5 frames. This procedure produced a final dataset comprising 58,740 individual data points, each containing the essential energy and force information derived from first-principles

calculations. This process is depicted in Figure S3a and Figure S3b.

Exploration. Molecular dynamics simulations were performed using the LAMMPS package[4] integrated with the Deep Potential (DeePMD) method to sample configuration spaces. During the exploration phase, we employed various adsorption configurations on both pristine and reconstructed surfaces as seed structures to conduct systematic sampling simulations. The DP-GEN sampling process consisted of multiple iterations, with model deviations evaluated based on the maximum force of the DP model to screen candidate configurations from MD trajectories. The trust level for configuration selection was defined by the lower and upper limits of model deviation, set to 0.10 eV/Å and 0.35 eV/Å, respectively. The setting of these thresholds, particularly the upper limit of 0.35 eV/Å, effectively identifies and filters out non-physical configurations, thereby preventing the sampling from entering unreliable regions and ensuring the robustness of the training process. Furthermore, our choice of force-deviation thresholds is consistent with those used in several published high-quality studies in related fields[5, 6]. Configurations falling within this range were selected for subsequent DFT labeling to improve the accuracy of the DP model in the next iteration. The sampling simulations were conducted under NVT ensemble conditions at temperatures of 300 K and 330 K. Each simulation ran for 15,000 steps, with a trajectory output frequency of 100 steps. Finally, a total of 40,193 configurations were labeled and incorporated into the final dataset.

Labeling. All density functional theory (DFT) calculations used the Vienna ab initio simulation package (VASP)[7]. The core electrons were treated with the Projector Augmented Wave (PAW) potential[8, 9], while the exchange-correlation functional was described within the generalized gradient approximation (GGA) using the revPBE functional[10]. A plane-wave kinetic energy cutoff of 400 eV was employed. The Brillouin zone was sampled using a Γ-centered k-point mesh with a KSPACING[11] of 0.3 Å^{-1}. The electronic self-consistent field (SCF) iterations employed a normal diagonalization algorithm with an energy convergence criterion of

1×10^{-5} eV. The DFT-D3 method with Becke-Jonson damping was included to account for van der Waals interactions[12].

Training. We trained corresponding Deep Potential (DP) models using the initial dataset and newly labeled configurations from the exploration stage. Four independent DP models were trained simultaneously per iteration via the DeePMD-kit package[13]. The embedding and fitting nets were pick to three hidden layers with (25, 50, 100) and (240, 240, 240) neurons, respectively. The cut-off radius was set to 7 Å. The hyperparameters start_pref_e, limit_pref_e, start_pref_f, and limit_pref_f represent the weighting coefficients for the energy and force terms in the total loss function. These were configured to increase from 0.02 to 1.0 for energy, and decrease from 1000 to 1.0 for forces, respectively. The learning rate followed an exponential decay schedule, starting from 0.001 and decaying every 1000 steps. In each DP-GEN iteration, the DP model was trained for 200,000 steps. After the DP-GEN process converged, the final effective models were further trained for another 200,000 steps under the same training configuration.

Given that our force field does not account for the deep structural reconstruction of interfaces (e.g., Cu atom agglomeration and bulk deep embedding of *H), the model's predictions may be uncertain or less accurate in such scenarios, and further validation via first-principles calculations is therefore recommended.

Grand Canonical Monte Carlo Simulation

Grand Canonical Monte Carlo (GCMC) simulations were employed to efficiently sample equilibrium configurations of *CO and *H co-adsorbed on Cu surfaces, through a statistical mechanics framework[14]. This approach generates history-dependent trajectories, enabling analysis of both final adsorbed structures and dynamic adsorption processes. All simulations were performed under conditions of constant chemical potential (μ), volume (V), and temperature (T).

At each Monte Carlo step, the system exchanges particles with external reservoirs through three types of trials: move, insertion, and remove. The acceptance probability

for each trial is given by:

Move trial:

$$P_{\text{move}} = min(1, \text{e}^{-\frac{\Delta U}{k_\text{B}T}})$$

where ΔU is the potential energy change after the trial move and k_B is the Boltzmann constant.

Insertion trial:

$$P_{\text{insert}} = min\left(1, \frac{V}{(N+1)\Lambda^3}\text{e}^{-\frac{\Delta U-\mu}{k_\text{B}T}}\right)$$

where V is the system volume, N is the number of particles in the system and Λ is the thermal de Broglie wavelength as $\frac{h}{\sqrt{2\pi m k_\text{B}T}}$.

Remove trial:

$$P_{\text{remove}} = min\left(1, \frac{N\Lambda^3}{V}\text{e}^{-\frac{\Delta U+\mu}{k_\text{B}T}}\right)$$

This study focuses on the co-adsorption of CO and H on Cu surfaces with fixed values of μ_{CO}, μ_H, V, T and N_{Cu}. The chemical potentials of H and of CO, μ_H and μ_{CO}, are calculated by

$$\mu_H(pH, U, T) = \frac{1}{2}E_{H_2}^{gas} - \ln(10)\, k_B T pH - |e|U_{SHE} + \left(ZPE^{gas} + C_p^{gas} - TS^{gas}\right) - (ZPE^{ads} + C_p^{ads} - TS^{ads})$$

$$\mu_{CO}(p_{CO}, T) = E_{CO}^{gas} + k_B T ln\frac{p_{CO}}{p^0} + \left(ZPE^{gas} + C_p^{gas} - TS^{gas}\right) - (ZPE^{ads} + C_p^{ads} - TS^{ads})$$

where the pH- and potential-dependent terms (with the electrode potential referenced to the SHE scale) are calculated using the computational hydrogen electrode (CHE) model; the zero-point energy (ZPE) and thermal contributions of the adsorbates are obtained from vibrational frequency calculations and evaluated at 298.15 K. The Boltzmann constant is k_B = 8.61733 × 10^{-5} eV K^{-1}, with pH = 7 and a CO partial pressure of p_{CO} = 1 bar. To ensure physical realism and consistency with experimental

conditions, the maximum *H coverage was constrained to 1 monolayer, corresponding to the full occupation of available hollow sites on the Cu surface.

To enhance sampling efficiency beyond conventional GCMC, we introduced structural relaxation using MLIPs after each Monte Carlo step, significantly improving phase space exploration while maintaining thermodynamic consistency. Accordingly, the algorithm employed in this work is a modified GCMC approach incorporating local optimization, which can also be referred to as Grand Canonical Basin Hopping (GCBH).[15-17]. Due to the basin-collapsing nature of the GCBH algorithm, configurations preferentially relax toward low-energy minima during the search process. As a result, the trajectory is more suitable for identifying low-energy metastable structures rather than strictly reproducing the thermodynamic probability distribution of a grand-canonical ensemble. In the present study, the statistical analysis is used to identify robust structural trends across the sampled structures.

GC-DFT

Under a constant applied potential, the electrode surface effectively constitutes a grand canonical ensemble of electrons, in which the number of electrons adjusts to accommodate changes in the surface work function. The potential-dependent electronic grand canonical free energy of the surface, Ω_{el}, can be approximated using a surface charging model[18]

$$\Omega_{el}(U) = E(U) - q(U) \cdot FU \approx E(U_0) - \frac{1}{2}C(U - U_0)^2$$

which treats the electrochemical interface as an equivalent capacitor. E(U) represents the electronic energy of the surface at potential U, calculated by referencing the Fermi level of the system relative to the vacuum level. q(U) denotes the surface charge difference relative to the neutral system, and F is Faraday's constant. U_0 stands for the potential of zero charge on the vacuum scale, and C is the effective capacitance.

Through systematic variation of the electron count in the system, the electronic energy E(U) and corresponding surface charge q(U) at a given potential U can be

determined. This allows for the sampling of a series of charge values to fit a quadratic relationship between the electronic grand canonical free energy $\Omega_{el}(U)$ and the potential U. The potential U, expressed on the vacuum scale, can be converted to the standard hydrogen electrode (SHE) scale using a benchmarked value (4.44 V for VASPsol).[19]

Kinetic Modeling

For the GCMC simulation results, we performed statistical analyses and selected representative local structures for further evaluation. To more accurately assess the reaction kinetics, we carried out DFT-based nudged elastic band (NEB) calculations on these configurations.

Hydrogen Migration

To investigate the migration behavior of hydrogen atoms on copper-based surfaces, periodic slab models with four different crystal facets were constructed, including 4-layer 3×3 Cu(100), 4-layer 3×3 Cu(111), 4-layer 1×3 Cu(211), and 4-layer 1×3 Cu(711). The slab thickness has a minimal influence on the hydrogen migration barrier (Figure S22). In all calculations, the bottom two atomic layers were fixed to simulate the bulk environment. To further explore the metal doping effect, one surface Cu atom in the 4-layer 3×3 Cu(100) model was substituted with Zn, Al, or Ga atoms, and the H migration energy barriers were calculated under the same constraints. Additionally, to examine the influence of local structural reconstruction on H migration, several 3×3 local configurations were randomly extracted from the GCMC simulation results of the Cu(100) surface, and the energy barriers for *H migration from the surface to the subsurface were systematically evaluated.

Cu Adatom Formation

Using 3-layer 3×3 Cu (100), 3-layer 3×3 Cu (111), 3-layer 1×3 Cu (211), and 3-layer 1×3 Cu (711) surfaces as initial models, the formation behavior of Cu adatoms was investigated. For each surface, *CO molecules were adsorbed at top sites with varying

coverages of *H_{sur} and *H_{sub} introduced in the vicinity, corresponding to representative local configurations identified from the GCMC simulations. The formation energies of Cu adatoms were systematically evaluated to understand the effects of different surface structures and hydrogen environments.

CO Protonation

To examine the influence of different geometric coordination environments on the electrochemical *CO reduction pathway to *CHO, various surface models were constructed. These included the pristine 4-layer 3×3 Cu(100) surface, Cu(100) with one and two adsorbed Cu adatoms, the pristine 4-layer 3×3 Cu(111) surface, Cu(111) with one and two adsorbed Cu adatoms, as well as the step and terrace sites of 3-layer 1×3 Cu(211) and Cu(711) surfaces.

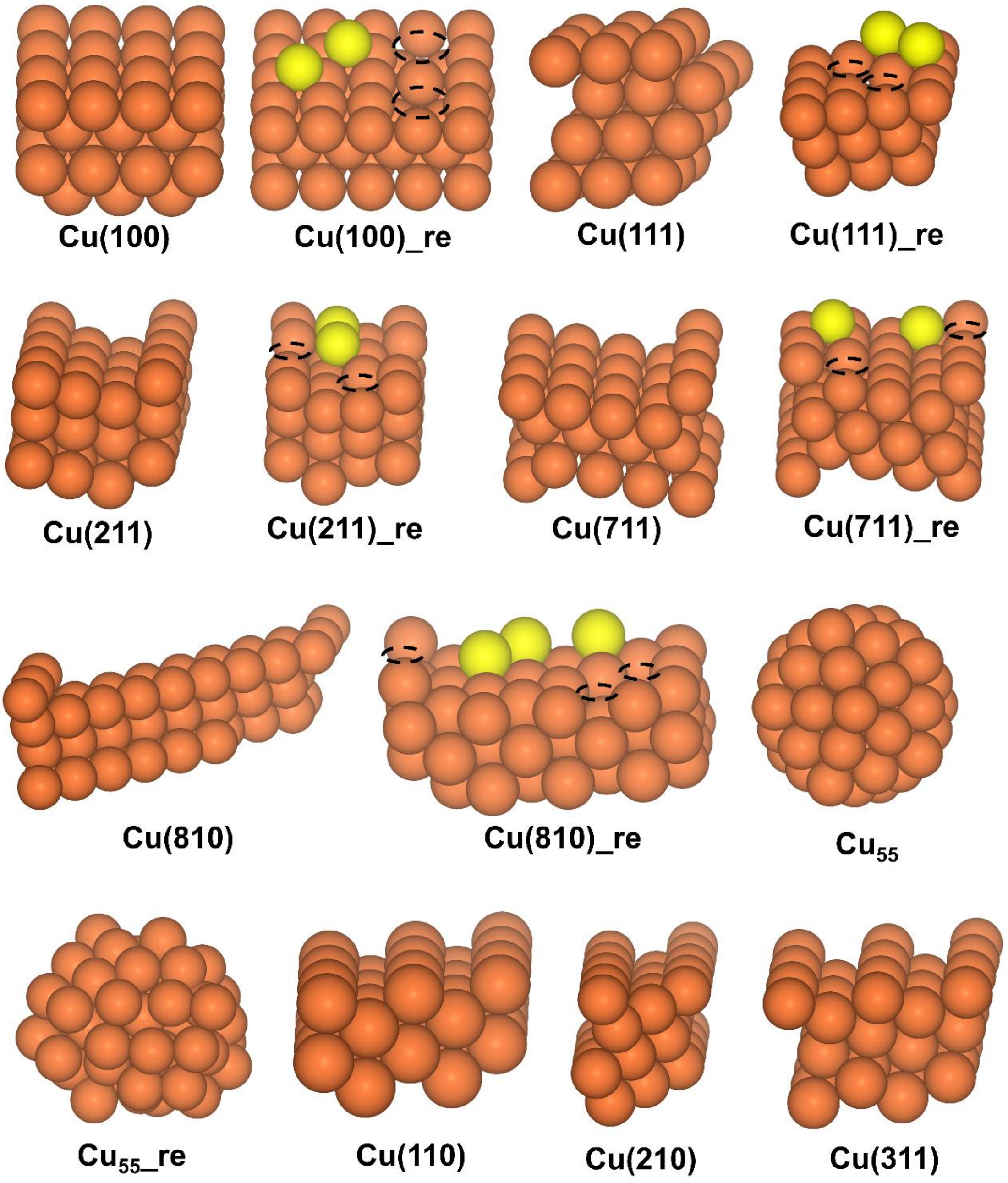

Figure S1. Initial surface configurations of the Cu catalyst (prior to adsorbate adsorption). Computational models of the pristine and reconstructed (labeled with "_re") surfaces selected for constructing the initial dataset. Color code: Cu (brown), Cu adatom (yellow), vacancy site (black dashed circle).

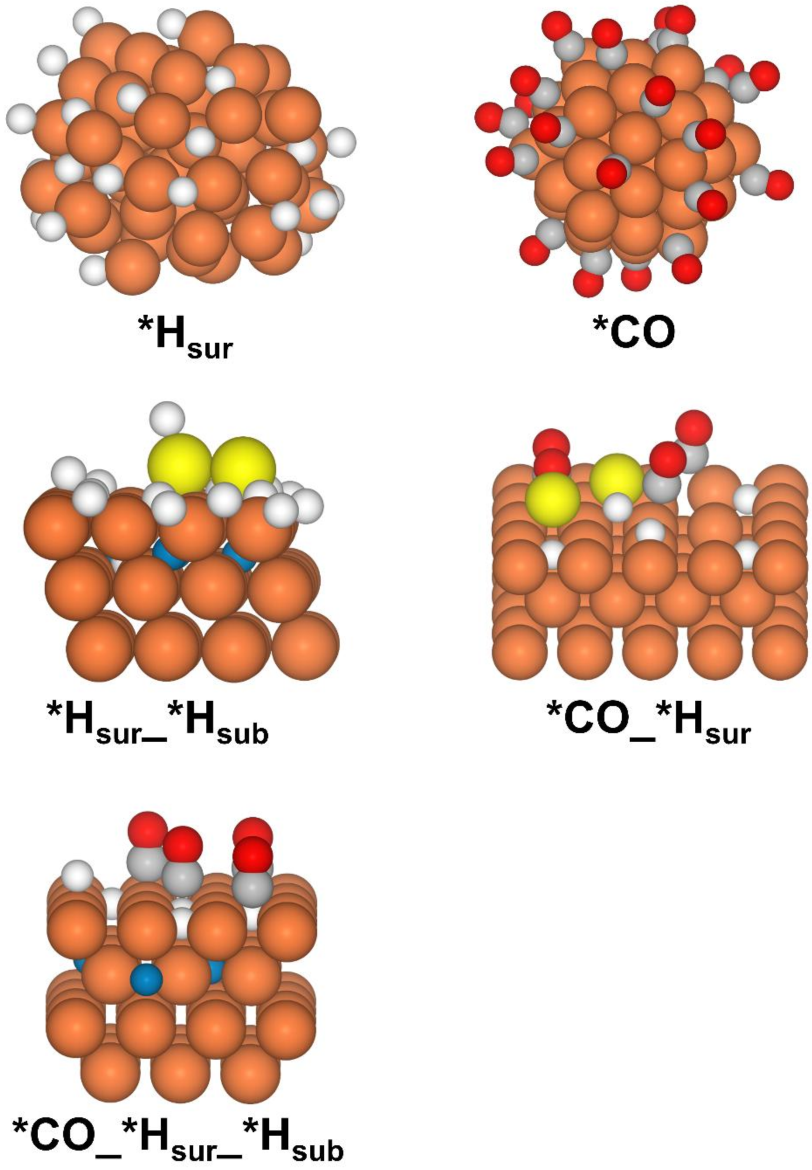

Figure S2. Representative Adsorption Configurations on the Cu Catalyst. Computational models illustrate the five categories of representative adsorption configurations for CO, $*H_{sur}$, and $*H_{sub}$ species investigated in this study. Color code: Cu (brown), Cu adatom (yellow), C (gray), O (red), H_{sur}(white), H_{sub}(blue).

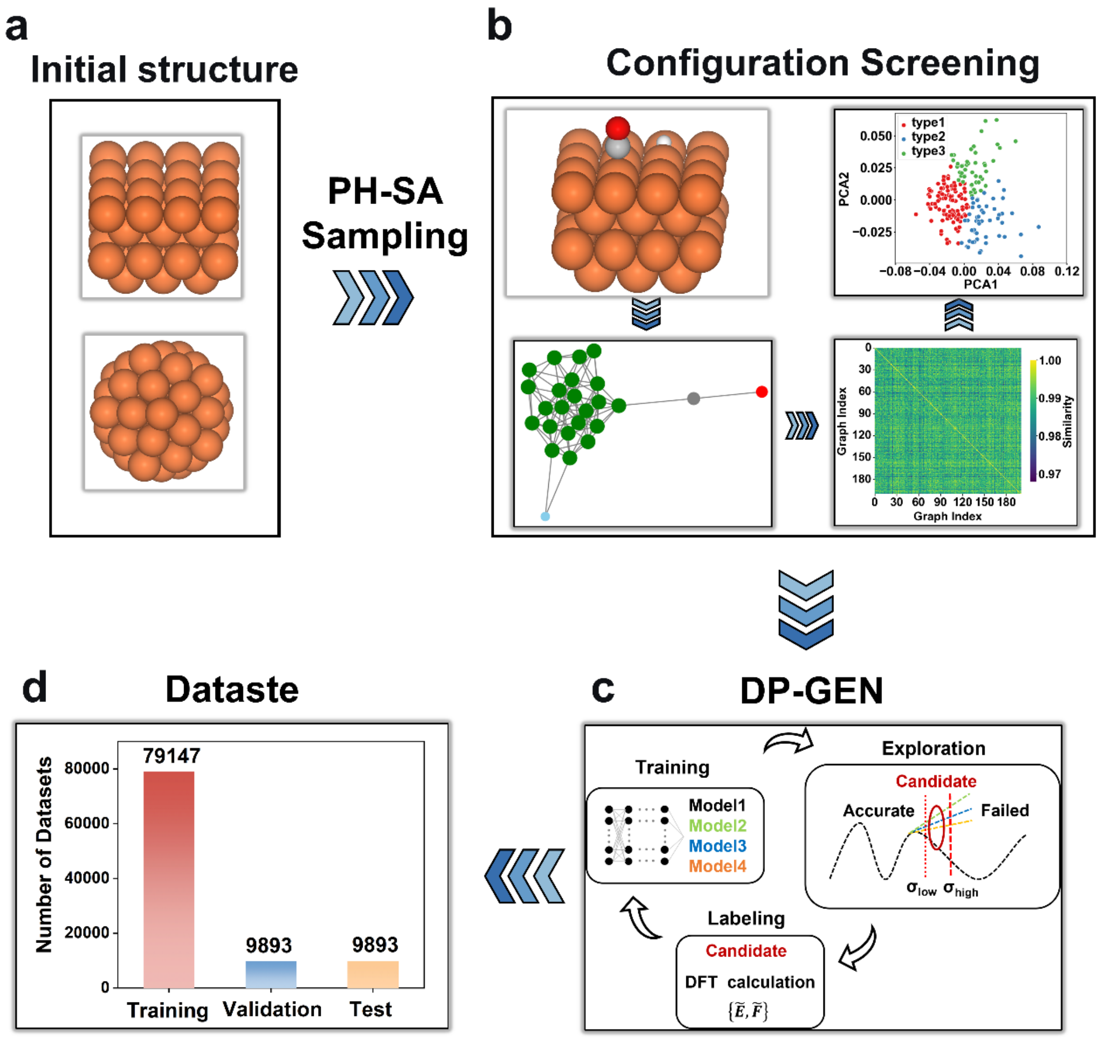

Figure S3. Workflow of configuration sampling and dataset construction. (a) Initial configuration; Large-scale configuration sampling via PH-SA method; (b) Screening of generated configurations through their conversion into node graphs, followed by similarity matrix computation and principal component analysis (PCA) for dimensionality reduction, leading to the identification of representative initial configurations; (c) Schematic of the DP-GEN active learning procedure; (d) Composition of the final dataset, split into training, validation, and test sets in an 8:1:1 ratio.

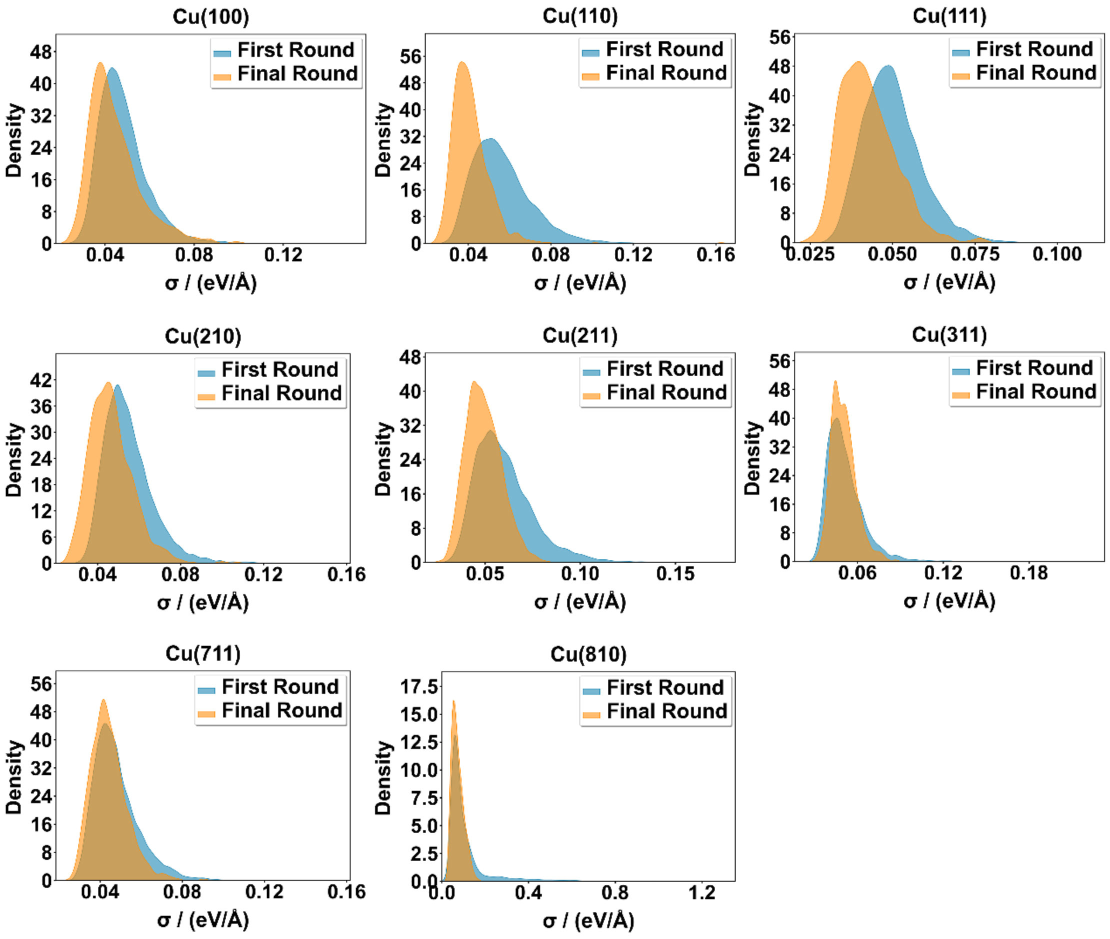

Figure S4. Evolution of maximum force deviation distributions across structural classifications during the active learning procedure. Panels display comparative density distributions for the initial and final rounds of the Cu–C–O–H dataset. The horizontal axis represents the standard deviation (σ) of force predictions among four independent MLIP models, while the vertical axis indicates the corresponding probability density. By the final round, over 90% of the explored structures displayed a force deviation (σ) of less than 0.1 $eV{\cdot}Å^{-1}$, enabling the Cu-C-O-H MLIPS to accurately capture the interatomic forces governing the co-adsorption and interactions of *CO and *H on Cu catalysts with DFT-level precision.

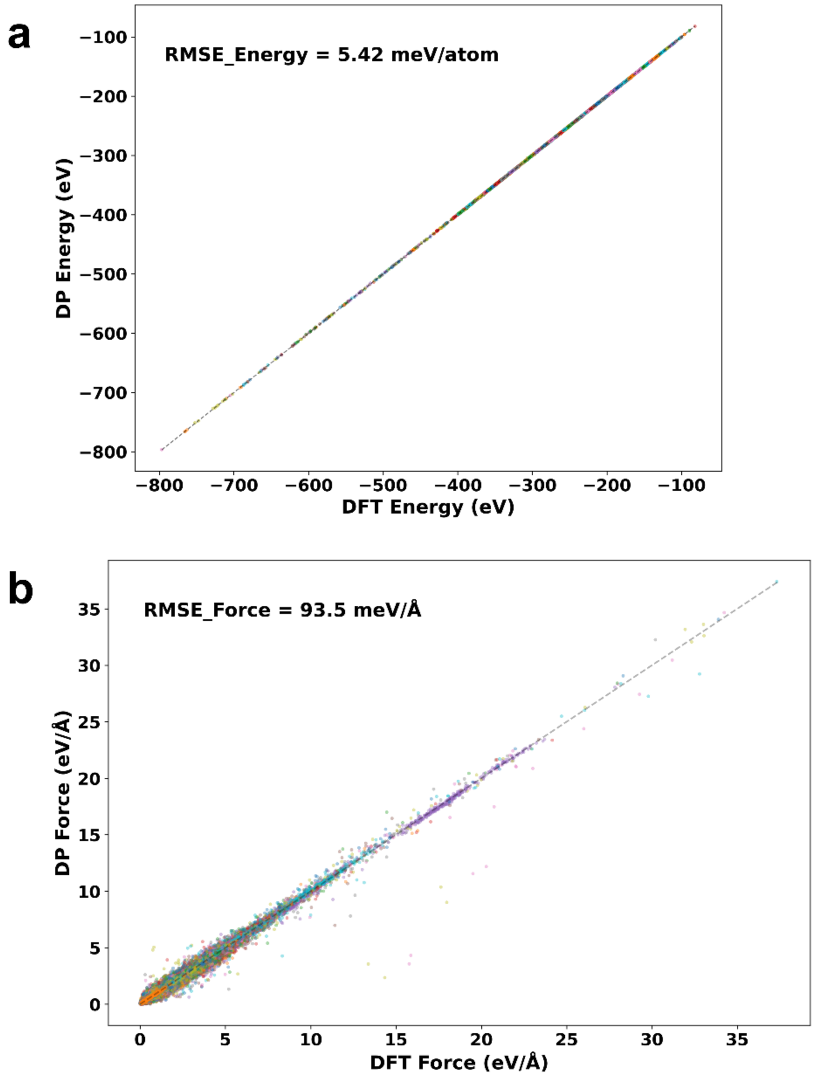

Figure S5. Validation of the Cu-C-O-H MLIPs against DFT. Comparison of the root-mean-square error (RMSE) for (a) energy (RMSE_Energy) and (b) force (RMSE_Force).

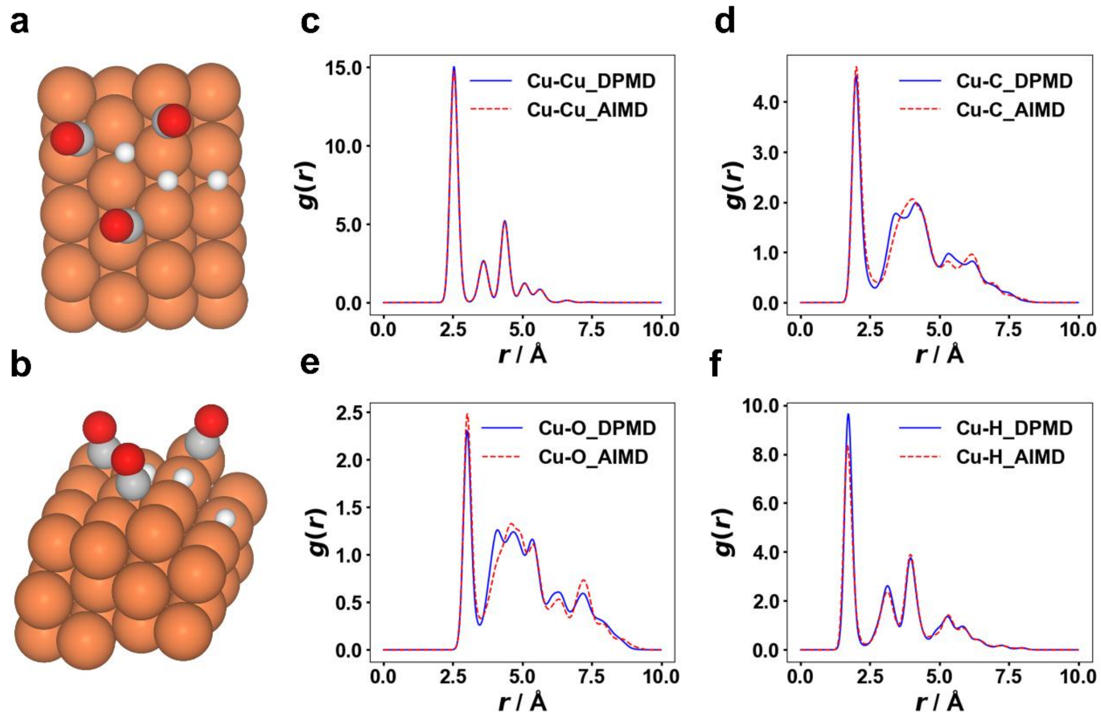

Figure S6. Structural Visualization and Validation of the MLIPs for Adsorbates on Cu(533). (a, b) Top and side views of the adsorption configuration with three CO and three H species. (c-f) Radial distribution functions (RDFs) of (c) Cu-Cu, (d) Cu-C, (e) Cu-O, and (f) Cu-H bonds, comparing AIMD and DPMD simulations. Color code: Cu (brown), C (gray), O (red). H (white).

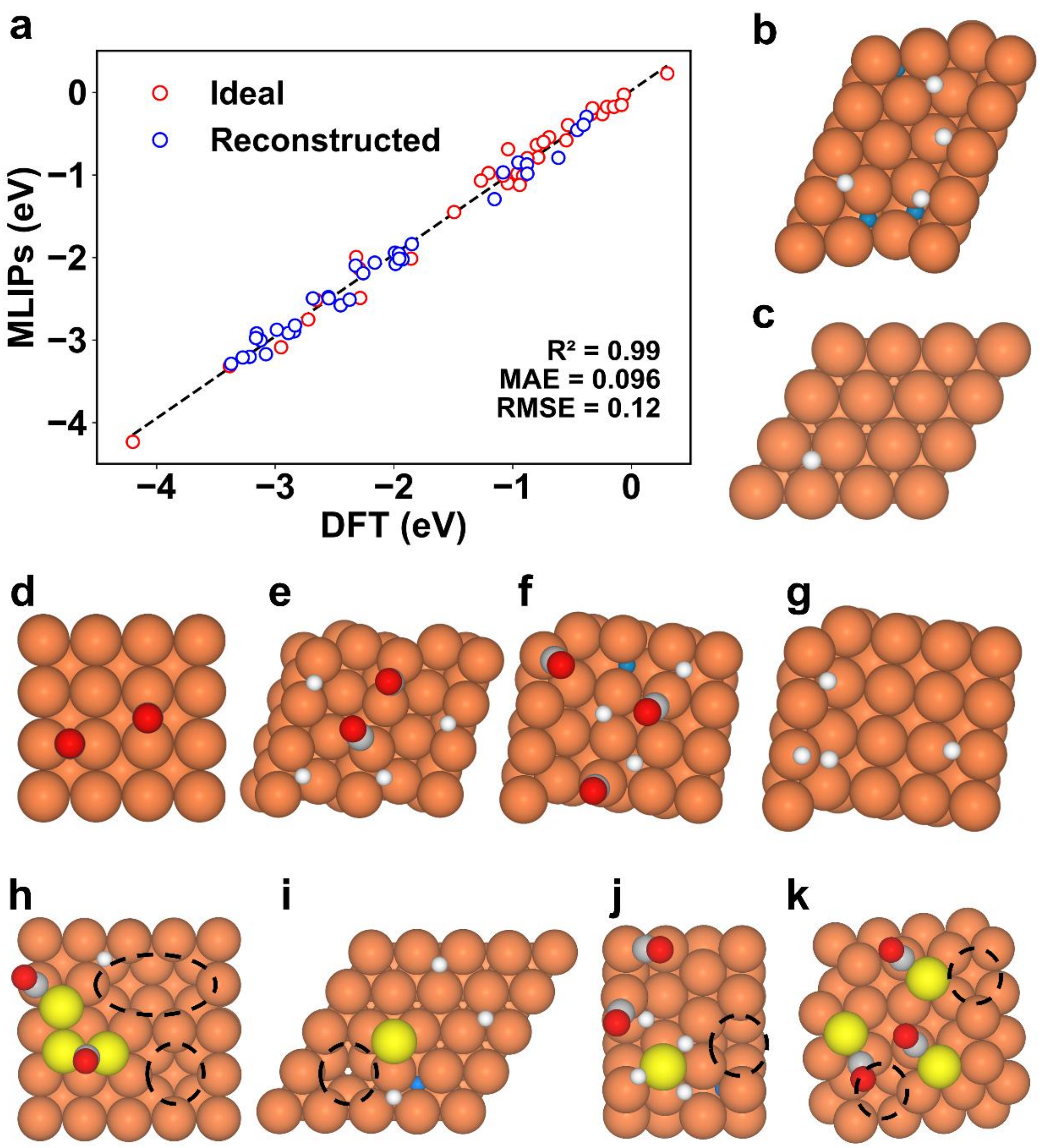

Figure S7. Comparison of adsorption energies for *CO and hydrogen species computed by DFT and the MLIP. (a) Correlation plot between DFT and MLIP calculated adsorption energies for *CO, *H_{sub}, and *H_{sur} on both reconstructed and ideal surfaces. (b–g) Representative atomic configurations of the adsorbed species on ideal surfaces, corresponding to the red data points in panel (a). (h–k) Representative atomic configurations of the adsorbed species on reconstructed surfaces, corresponding to the blue data points in panel (a). Color code: Cu (brown), Cu adatom (yellow), vacancy site (black dashed circle), H_{sur}(white), H_{sub}(blue).

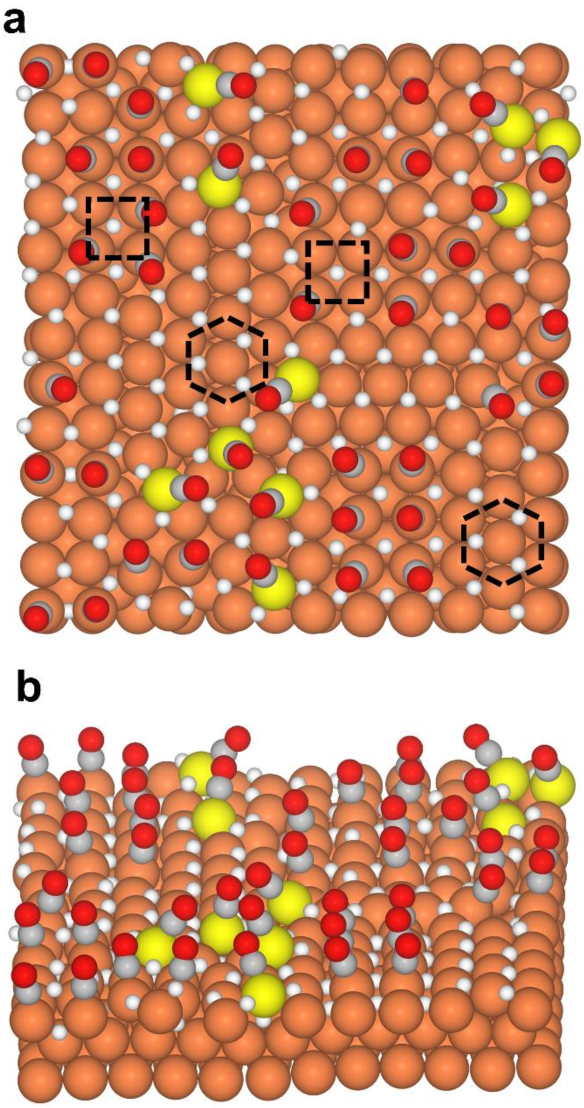

Figure S8. (a) Top view and (b) side view of the molecular configuration on the Cu(100) surface obtained from grand canonical Monte Carlo (GCMC) simulations under an applied potential of -1.0 V vs. RHE. Color code: Cu (brown), C (gray), O (red), H (white), Cu adatom (yellow).

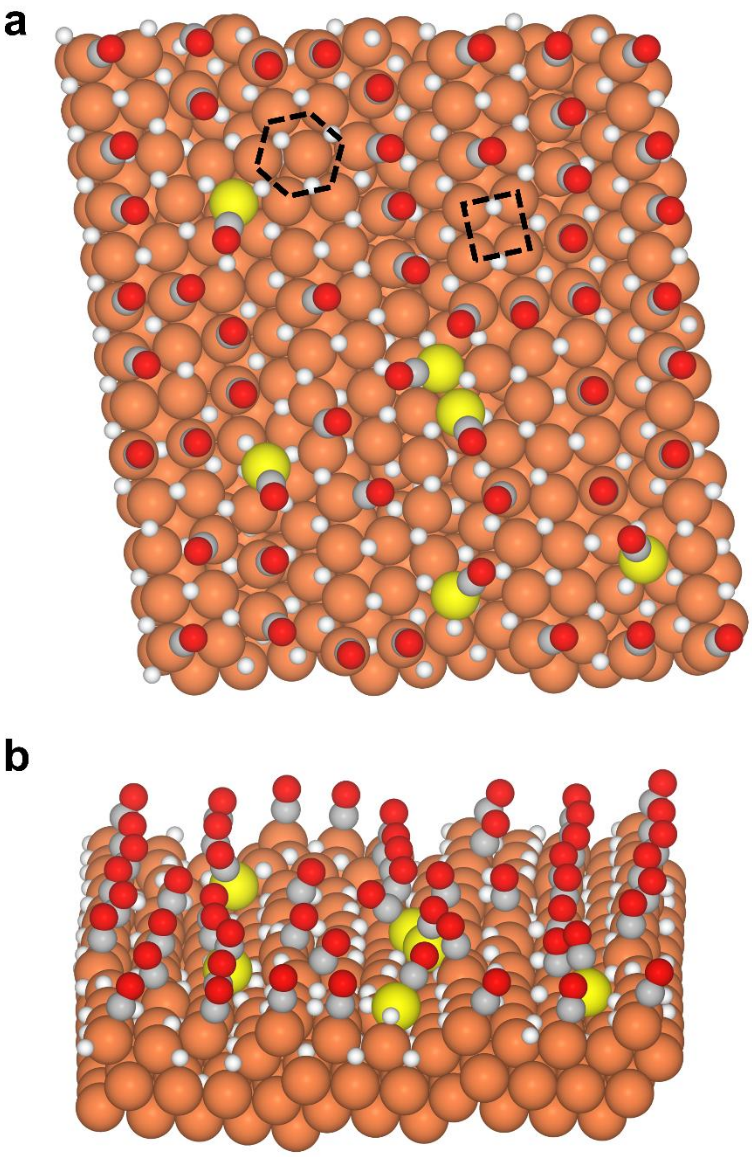

Figure S9. (a) Top view and (b) side view of the molecular configuration on the Cu(711) surface obtained from grand canonical Monte Carlo (GCMC) simulations under an applied potential of -1.0 V vs. RHE. Color code: Cu (brown), C (gray), O (red), H (white), Cu adatom (yellow).

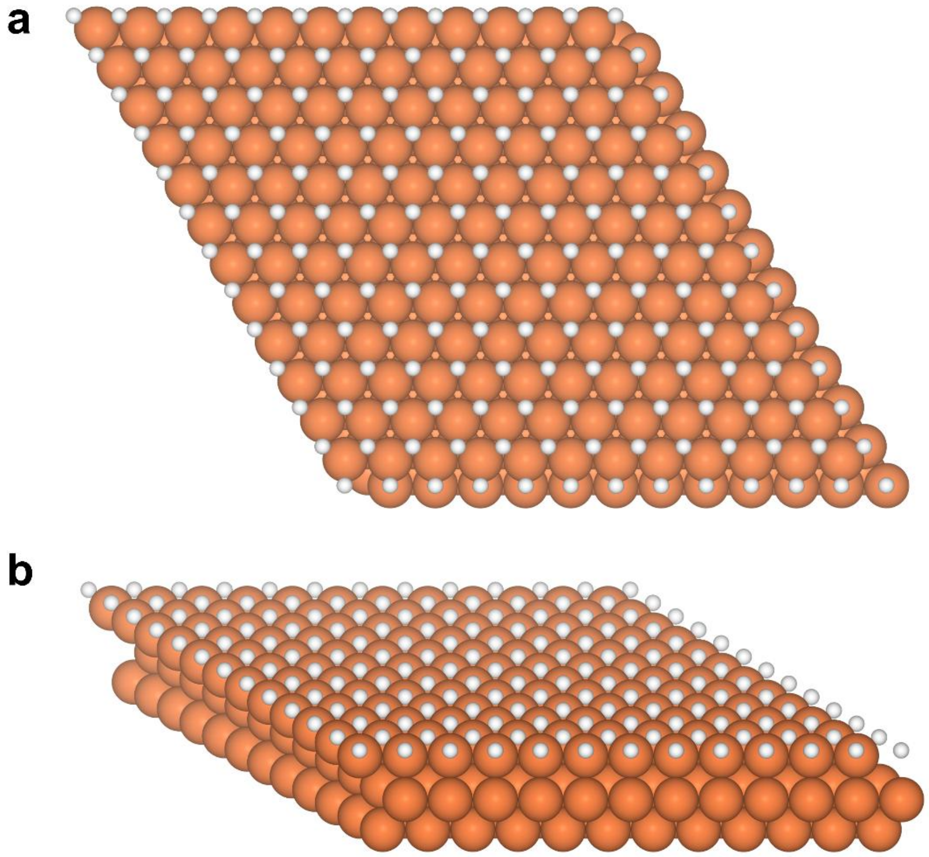

Figure S10. (a) Top view and (b) side view of the molecular configuration on the Cu(111) surface obtained from grand canonical Monte Carlo (GCMC) simulations under an applied potential of -1.0 V vs. RHE. Color code: Cu (brown), C (gray), O (red), H (white), Cu adatom (yellow).

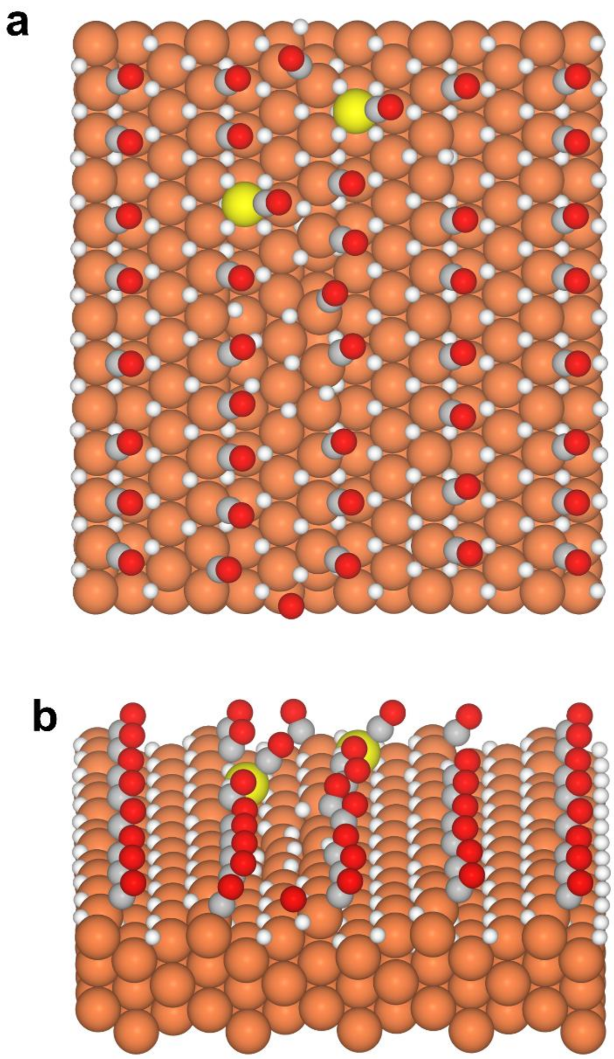

Figure S11. (a) Top view and (b) side view of the molecular configuration on the Cu(211) surface obtained from grand canonical Monte Carlo (GCMC) simulations under an applied potential of -1.0 V vs. RHE. Color code: Cu (brown), C (gray), O (red), H (white), Cu adatom (yellow).

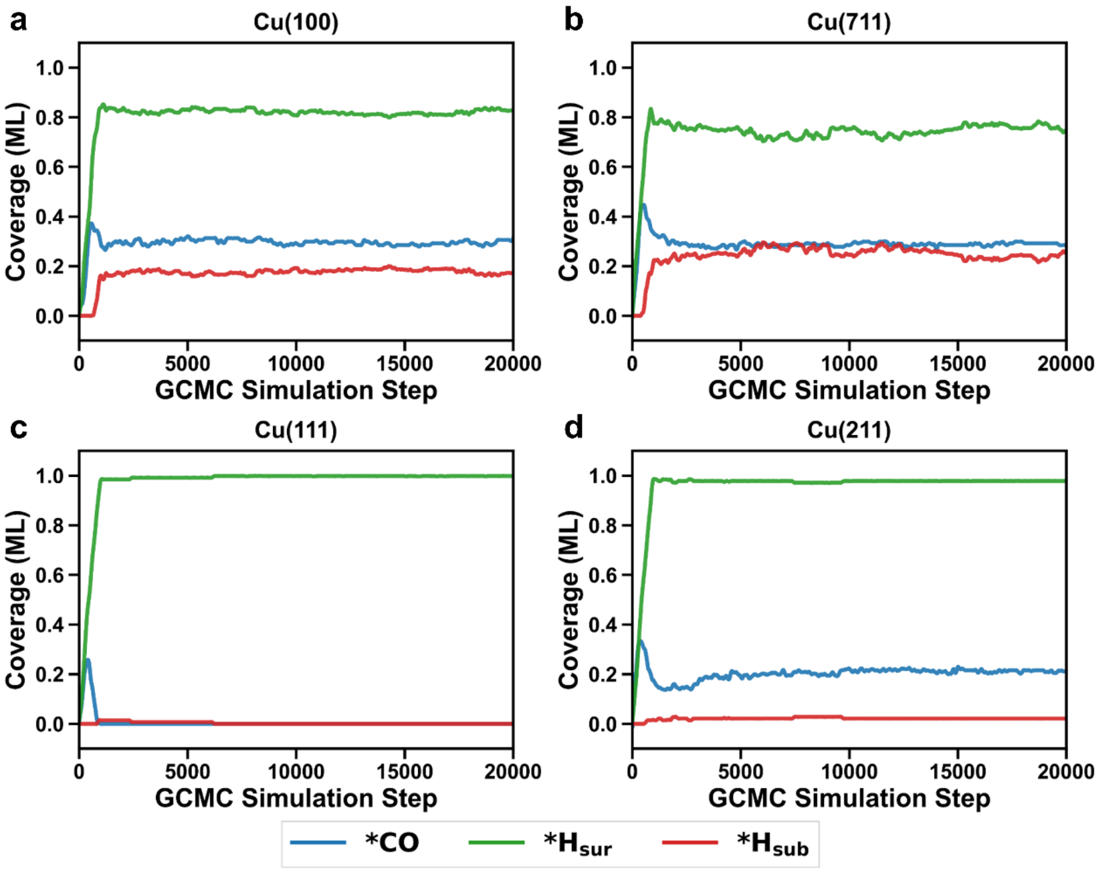

Figure S12. Evolution of surface species during the extended 20000 steps GCMC simulations. Coverage of *CO, *H$_{sur}$, and *H$_{sub}$ as a function of simulation steps on the (a) Cu(100), (b) Cu(711), (c) Cu(111), and (d) Cu(211) facets.

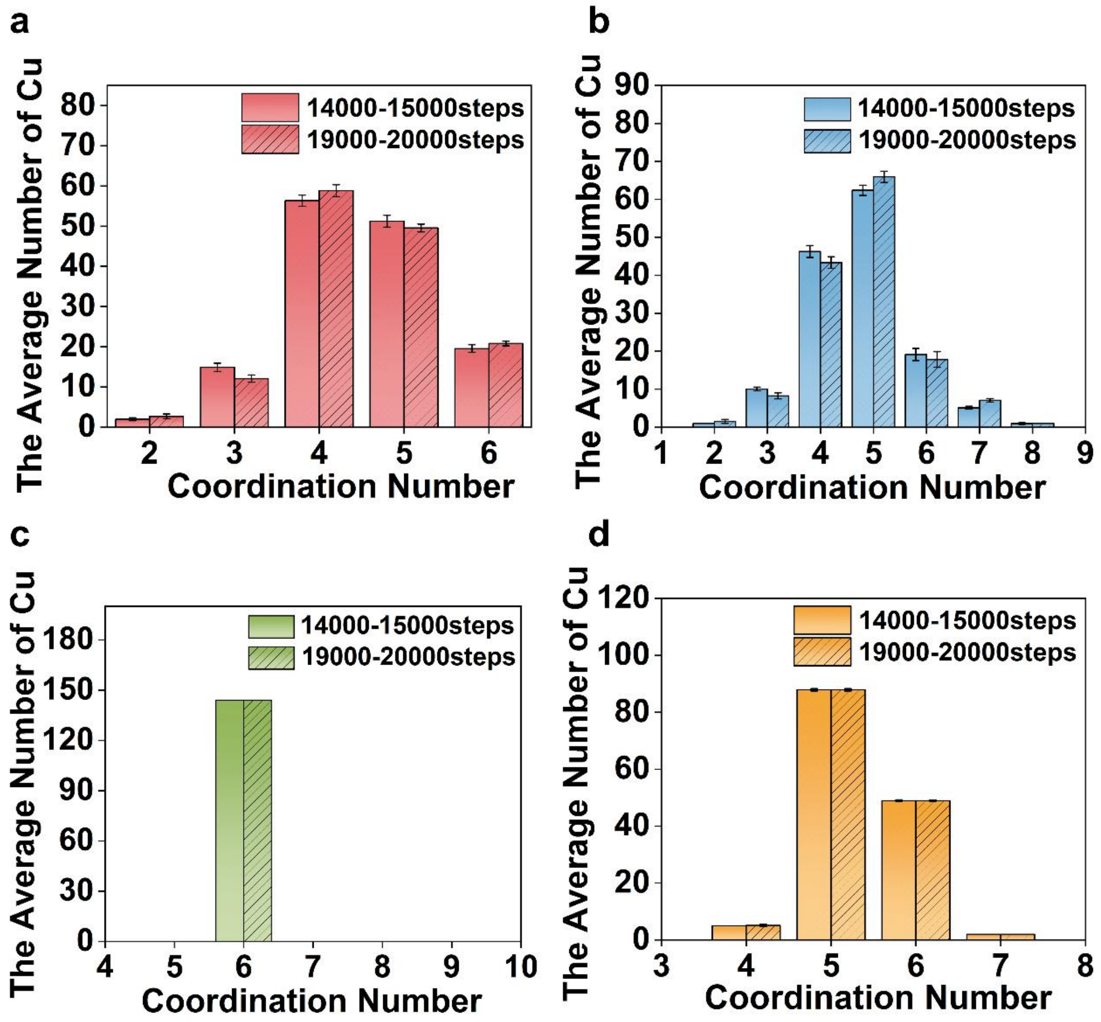

Figure S13. Statistical analysis of surface coordination environments. Comparative analysis of 50 evenly sampled configurations from the (a) Cu(100), (b) Cu(711), (c) Cu(111), and (d) Cu(211) facets during two simulation windows: 14,000–15,000 steps and 19,000–20,000 steps. Each panel presents the counts of atoms with different coordination numbers, with error bars indicating the standard deviation across sampled configurations.

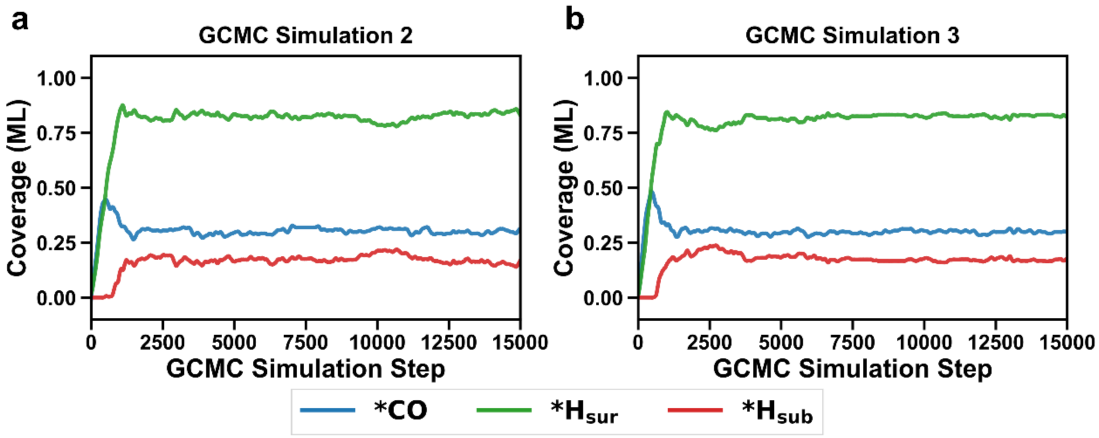

Figure S14. Additional independent grand canonical Monte Carlo (GCMC) simulations on the Cu(100) surface. Panels (a) and (b) present two complementary 15,000-step GCMC trajectories (GCMC Simulation 2 and GCMC Simulation 3, respectively), demonstrating the reproducibility of the coverage evolution trends observed in Figure 2a (GCMC Simulation 1).

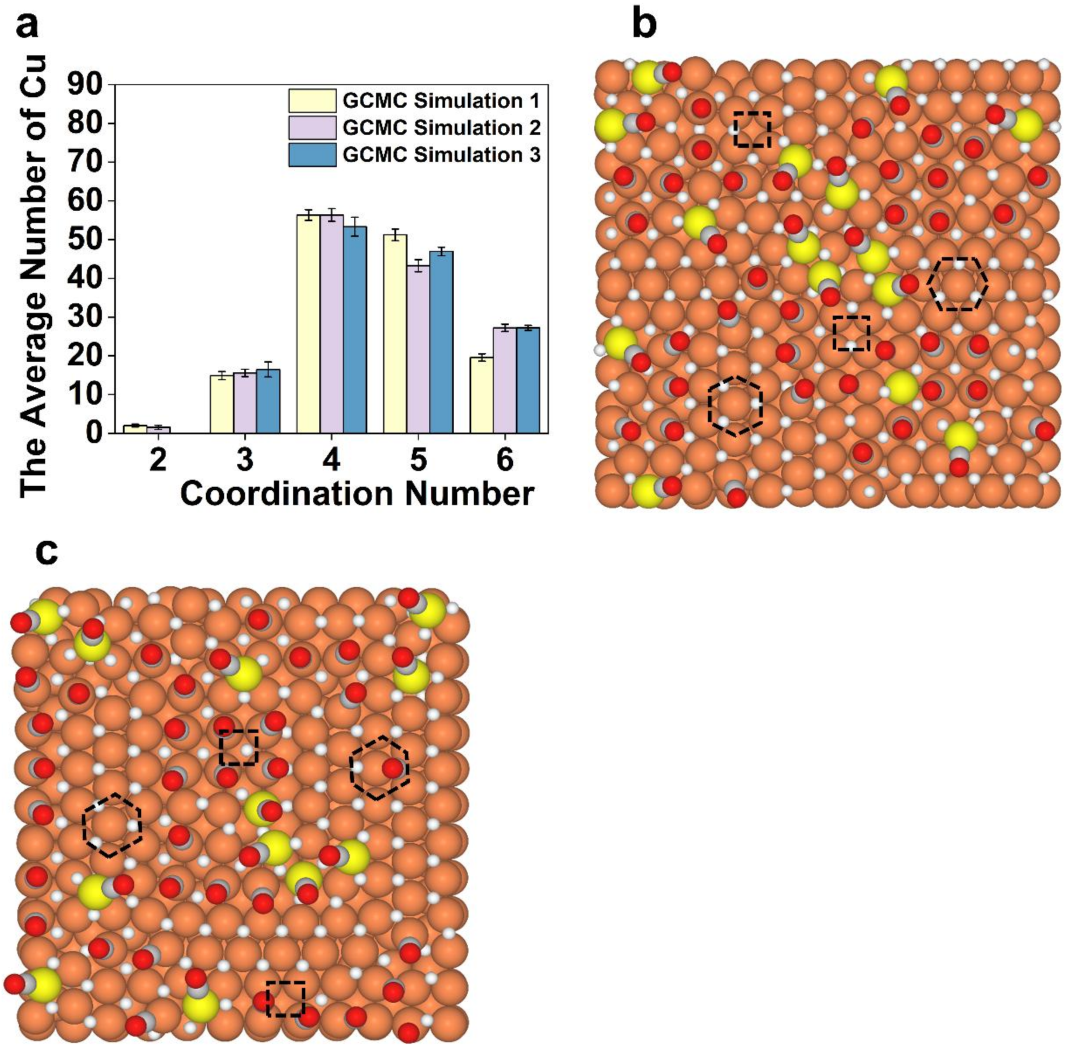

Figure S15. (a) displays the distribution of coordination numbers for surface atoms based on 50 configurations systematically extracted (every 20 steps) from the final 1000 frames of each of the three independent simulation trajectories. Panels (b) and (c) show the final configurations obtained from the GCMC Simulation 2 and GCMC Simulation 3, respectively.

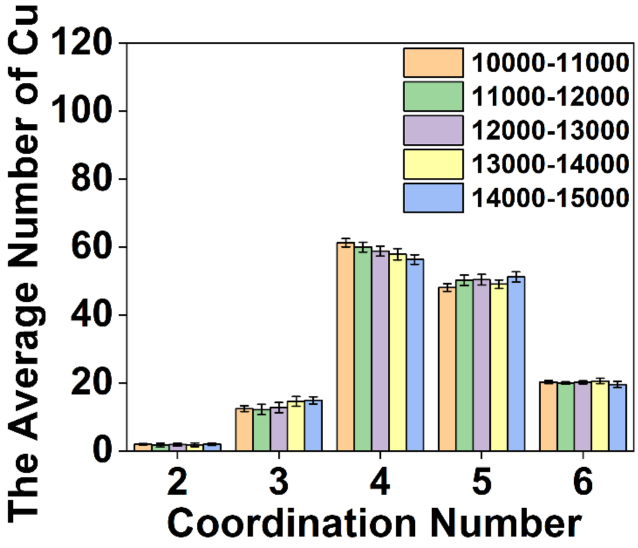

Figure S16. Analysis of coordination environments and associated errors for surface atoms, based on configurations sampled during different intervals of GCMC Simulation 1: 10000-11000, 11000-12000, 12000-13000, 13000-14000, and 14000-15000 steps. From each interval, 50 configurations were selected at intervals of every 20 steps for statistical evaluation. The analysis reveals that the coordination environments of surface atoms remain consistent across all sampled intervals, with only minor variations in the number of atoms assigned to each identical coordination state.

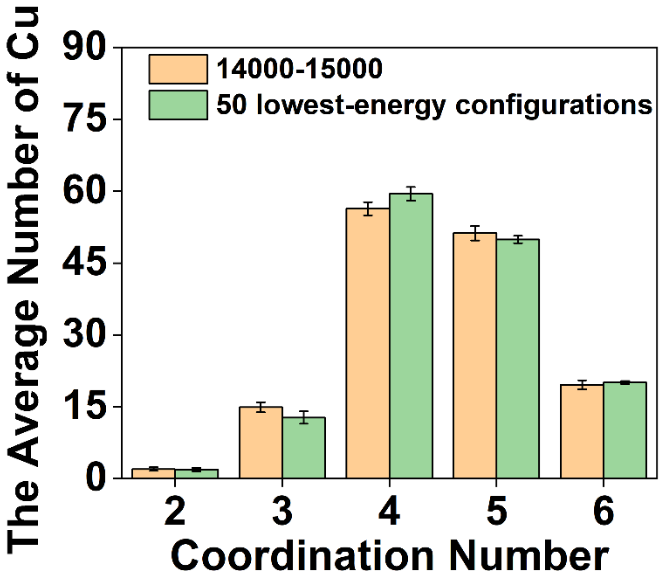

Figure S17. Coordination environment analysis and associated error estimation for surface atoms based on the trajectory from GCMC Simulation 1. Two independent statistical approaches were employed: (i) sampling the final 1000 steps of the simulation (14000-15000 steps) by selecting 50 configurations at intervals of every 20 steps; and (ii) selecting the 50 lowest-energy configurations from the entire 10000-15000 step simulation trajectory. The analysis demonstrates that both statistical methods yield identical surface coordination environments, with negligible differences in the population of atoms within each specific coordination state.

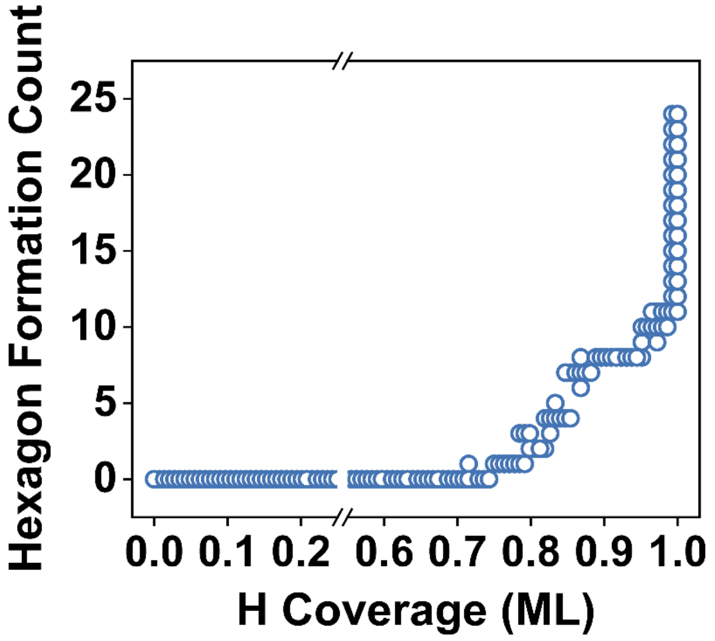

Figure S18. Distribution of hexagonal reconstruction domains on the Cu(100) surface as a function of hydrogen coverage during GCMC simulation.

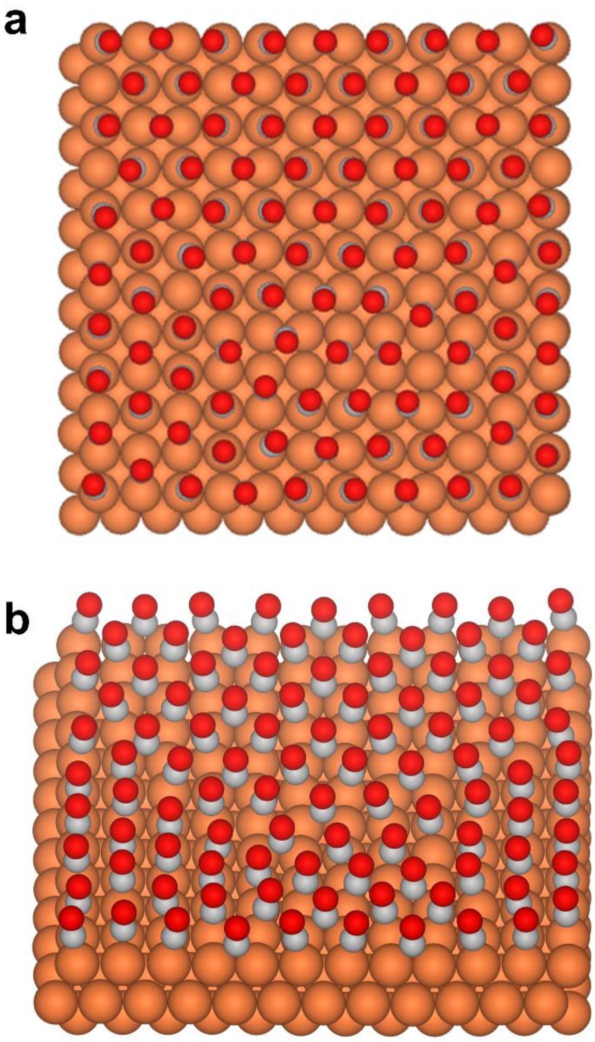

Figure S19. (a) Top view and (b) side view of the molecular configuration on the Cu(100) surface obtained from grand canonical Monte Carlo (GCMC) simulations under an applied potential of -0.2 V vs. RHE. Color code: Cu (brown), C (gray), O (red), H (white), Cu adatom (yellow).

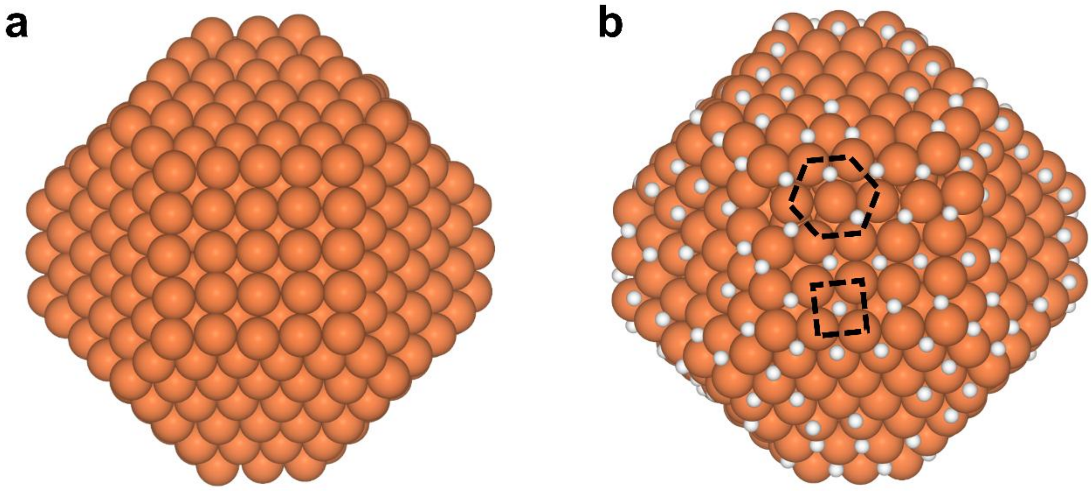

Figure S20. Comparative LAMMPS simulations of a copper nanocluster before and after hydrogen adsorption. (a) A ~2.5 nm Cu nanocluster was constructed based on the Wulff model by weighing the surface energies of relevant facets, resulting in a structure exclusively exposing (100) and (111) facets. A subsequent 20 ps molecular dynamics simulation prior to hydrogen adsorption confirmed that the (100) facets remained intact. (b) The equilibrated configuration of this structure after a further 20 ps simulation under high hydrogen coverage. Color code: Cu (brown), H (white).

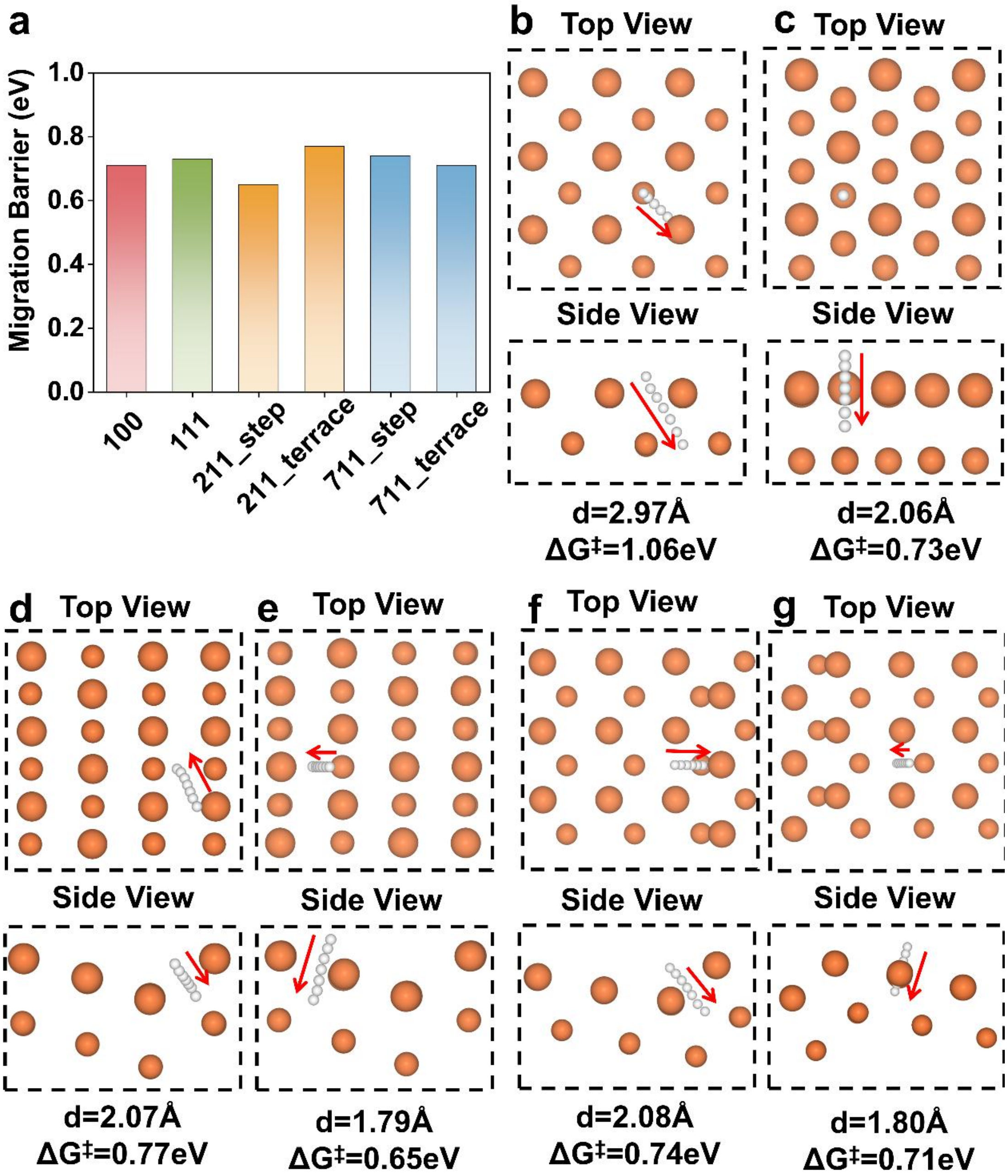

Figure S21. Energy Barriers and Pathways for Hydrogen Migration on Various Copper Facets. (a) Comparative energy barriers for hydrogen migration across different copper facets. (b, c) Atomic-scale migration pathways and corresponding energy profiles on flat Cu(100) and Cu(111) surfaces, respectively. (d, e) Site-dependent migration on the step and terrace sites of the Cu(211) surface. (f, g) Site-dependent migration on the step and terrace sites of the Cu(711) surface. Color code: Cu (brown), H (white).

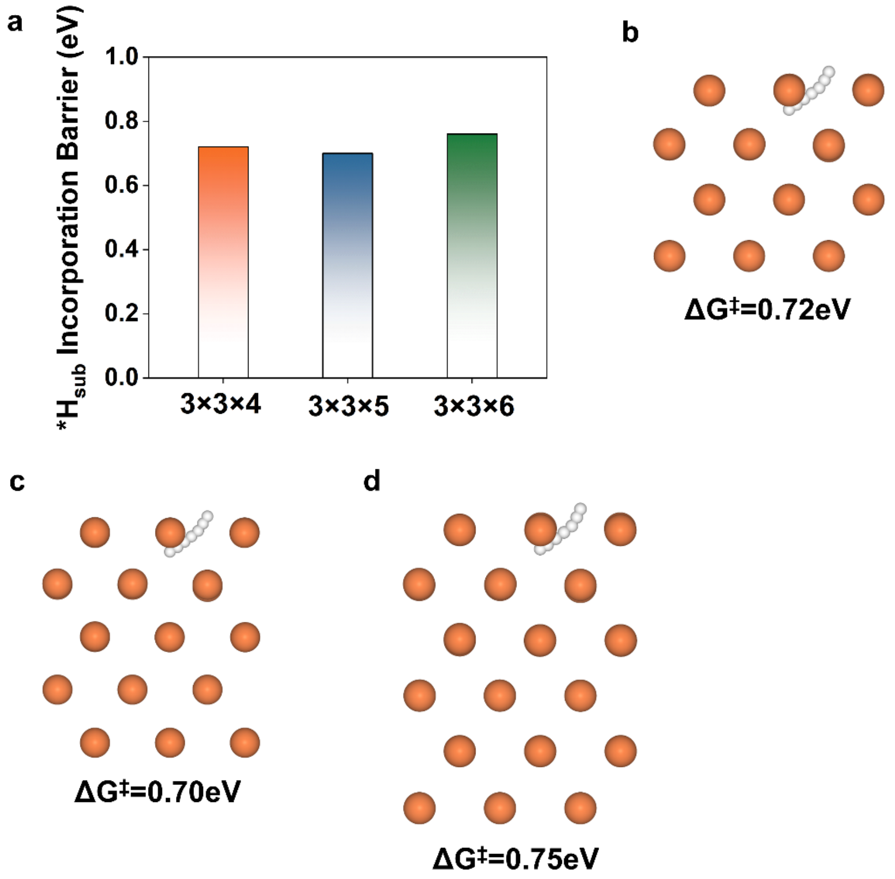

Figure S22. Energy barriers and schematic pathways for hydrogen migration into the subsurface on the Cu(100) surface. (a) Comparison of the calculated hydrogen migration barriers in slab models with different atomic-layer thicknesses (4, 5, and 6 layers). (b–d) Schematic migration pathways and corresponding energy profiles for hydrogen insertion into the subsurface in the 4-layer, 5-layer, and 6-layer slab models, respectively. Color code: Cu (brown), H(white).

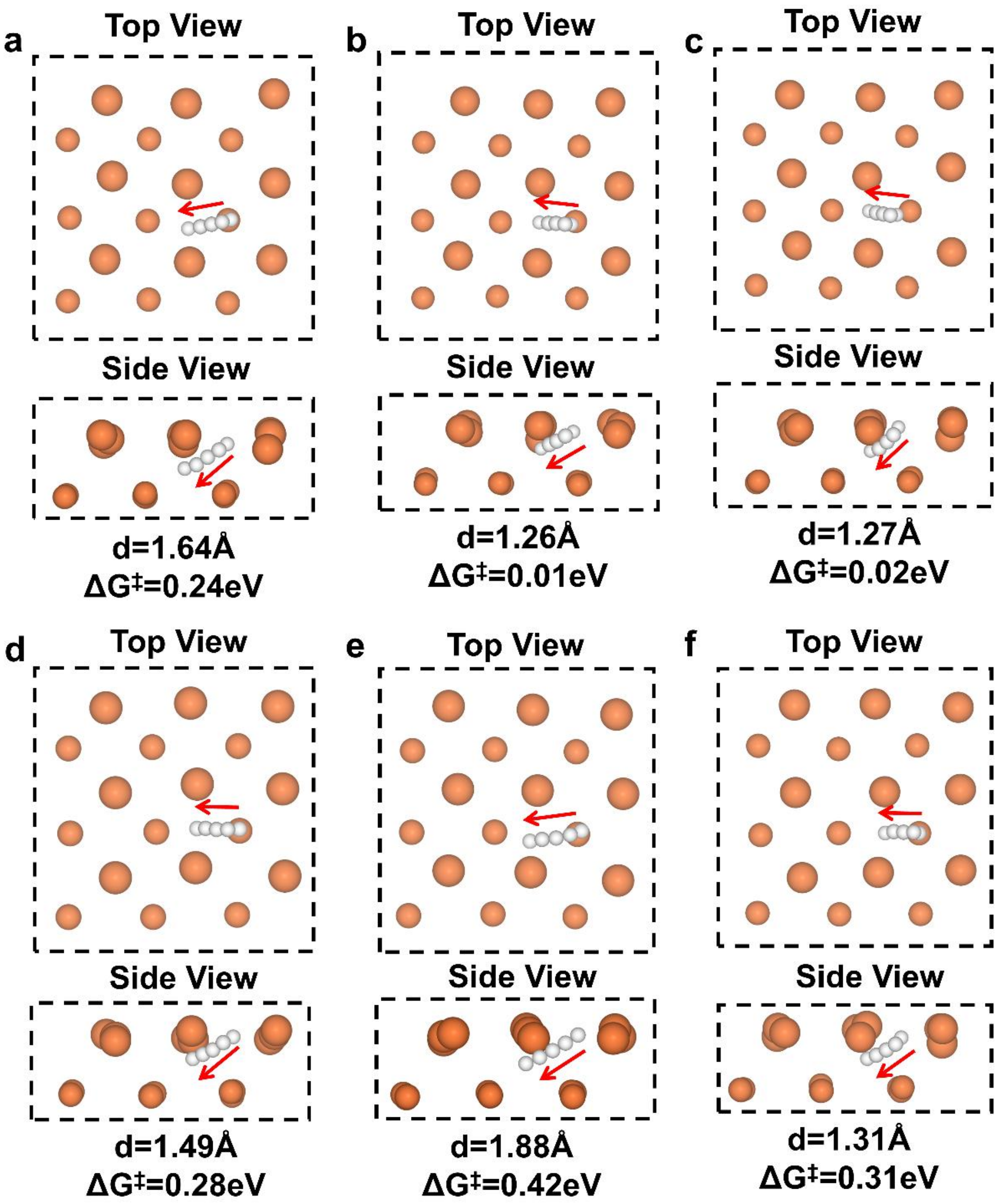

Figure S23. Six distinct local reconstruction environments (a-f) identified from the atomic configuration of the Cu(100) surface after GCMC simulation. Their corresponding hydrogen incorporation pathways and the associated energy barriers are determined. Color code: Cu (brown), H(white).

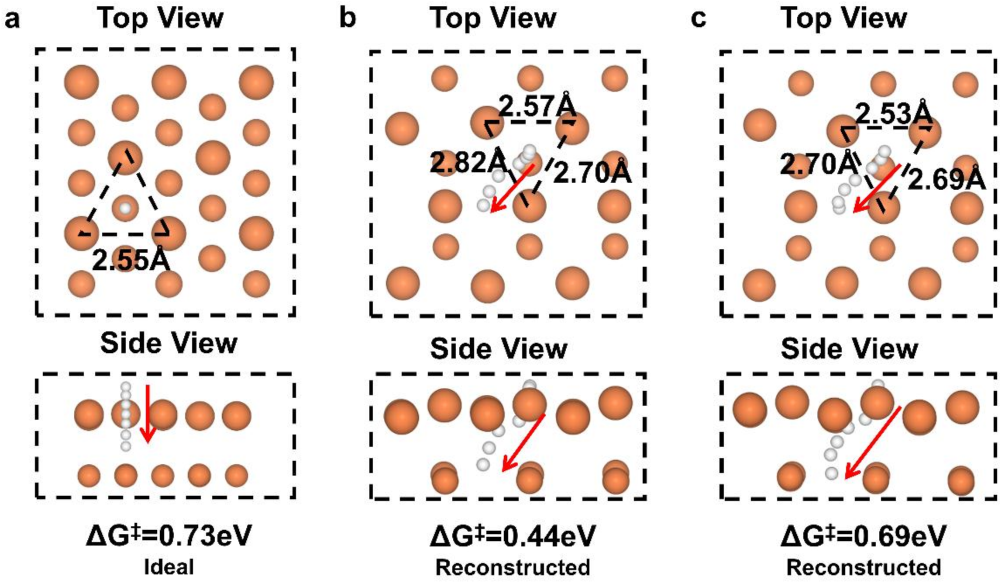

Figure S24. Comparison of hydrogen migration pathways and energy barriers from the surface to the subsurface at Cu(111) and reconstructed (111)-region of Cu(100). (a) Migration energy barrier, and schematic pathway for hydrogen insertion into the subsurface on an ideal Cu(111) surface. (b, c) Corresponding migration energy barriers, and schematic pathways for hydrogen insertion into the subsurface within the reconstructed (111)-like microenvironments on Cu(100). Color code: Cu (brown), H(white).

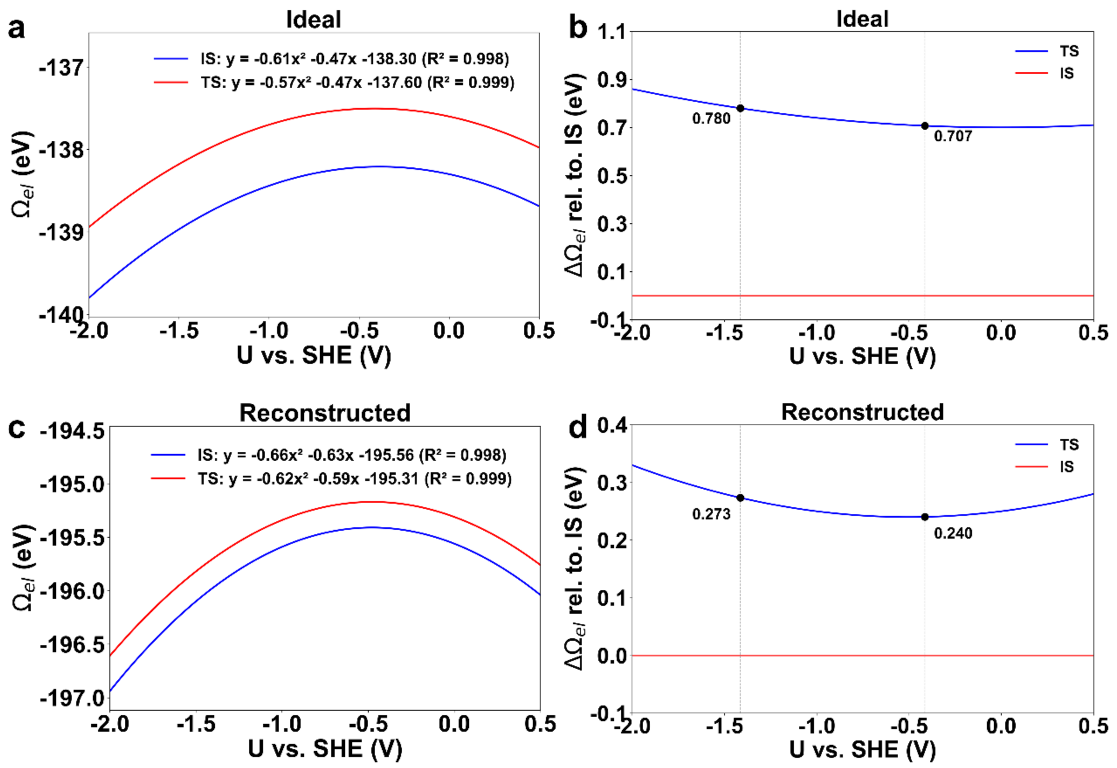

Figure S25. Grand-canonical DFT (GC-DFT) calculations at constant potential for the hydrogen migration pathways corresponding to (a, b) the ideal surface (Figure 3d) and (c, d) the reconstructed surface (Figure S23a). Panels (a) and (c) show the potential dependence (0 V to –1 V vs. RHE) of the free energy for the initial and transition states of the two pathways, respectively. Panels (b) and (d) present the corresponding migration barriers as a function of potential.

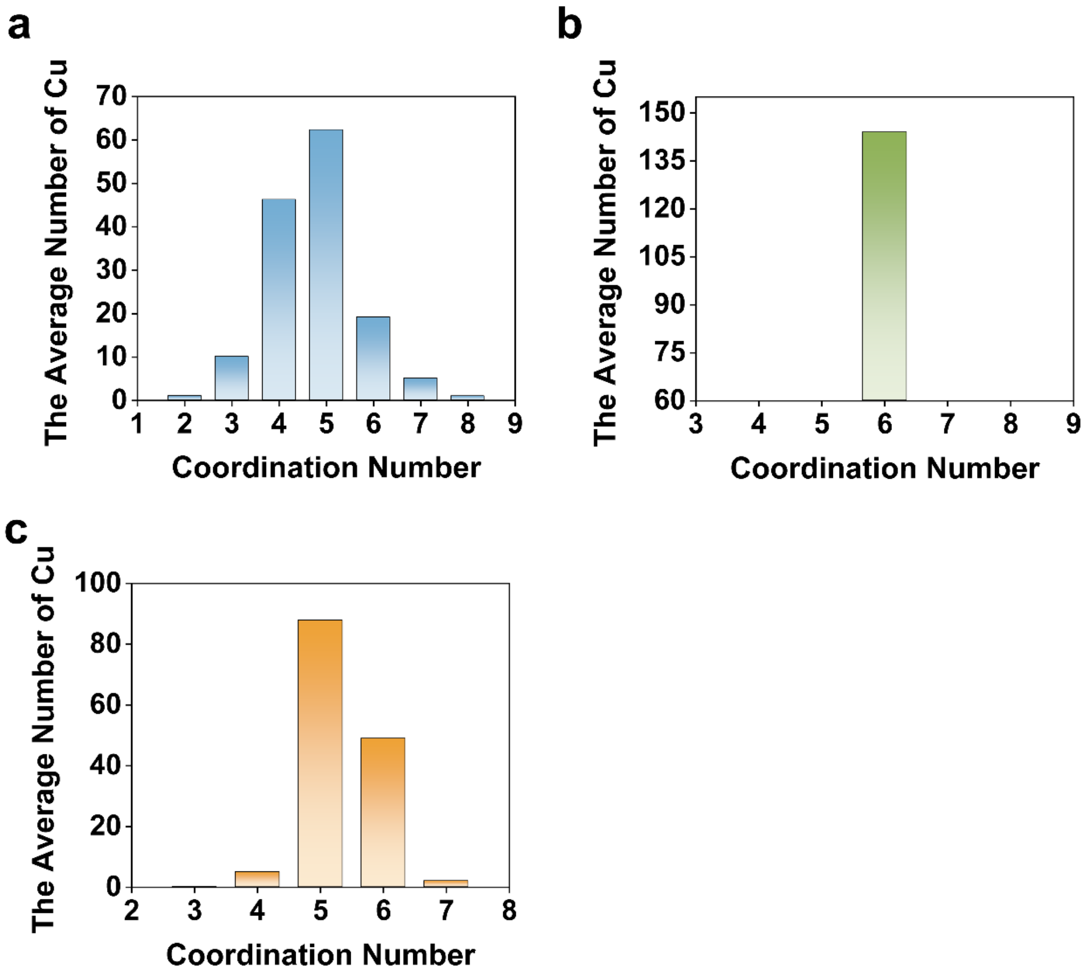

Figure S26. Statistical distribution of the average coordination numbers (CNs) of surface Cu atoms across three different facets. Data was extracted from 50 snapshots (every 20 frames from the last 1000) of GCMC simulations for (a) Cu (711), (b) Cu (111), and (c) Cu (211) surfaces.

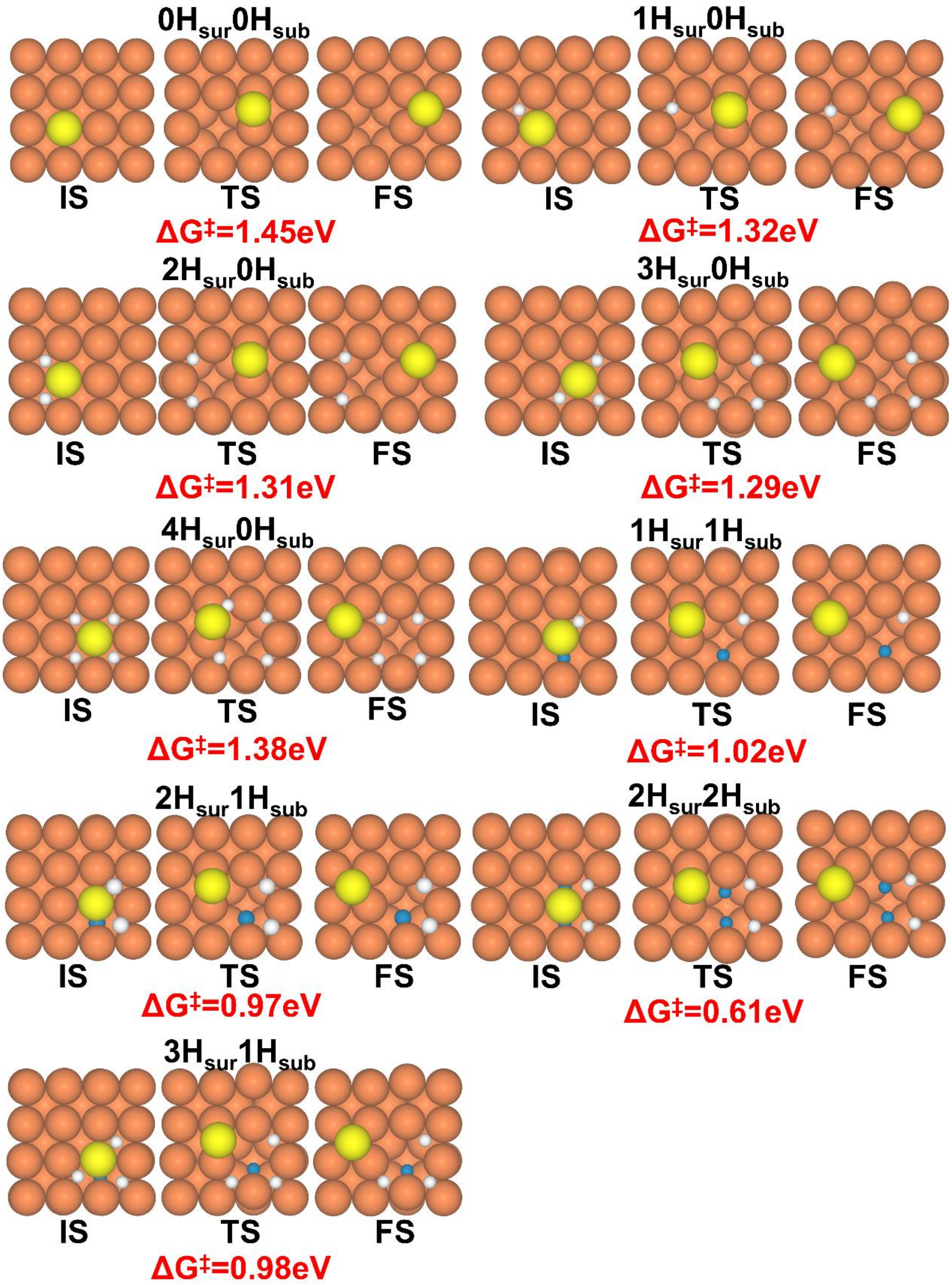

Figure S27. Atomic-scale pathways and energy barriers for Cu(100) Cu adatom formation in the absence of CO. Initial (IS), transition (TS), and final (FS) states are shown for Cu adatom formation under varying hydrogen coverages. Color code: Cu (brown), the dissolving Cu atom (yellow), H_{sur}(white), H_{sub}(blue).

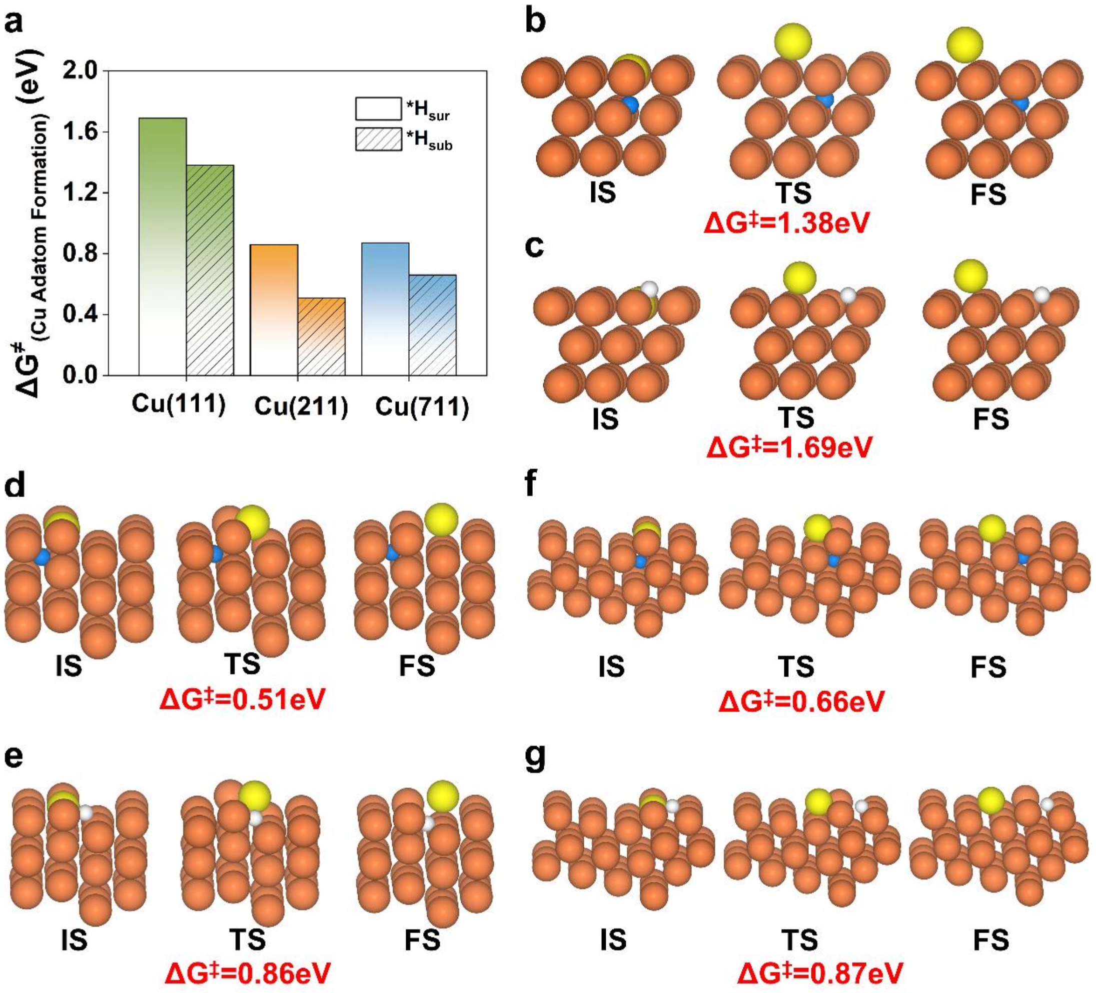

Figure S28. (a) Calculated energy barriers for Cu adatom formation on Cu(111), Cu(211), and Cu(711) facets in the presence of either a surface hydrogen (H_{sur}) or a subsurface hydrogen (H_{sub}). Schematics illustrating the formation of a Cu adatom: (b) with H_{sub} and (c) with H_{sur} on Cu(111); (d) with H_{sub} and (e) with H_{sur} on Cu(211); (f) with H_{sub} and (g) with H_{sur} on Cu(711). Color code: Cu (brown), the dissolving Cu atom (yellow), H_{sur}(white), H_{sub}(blue).

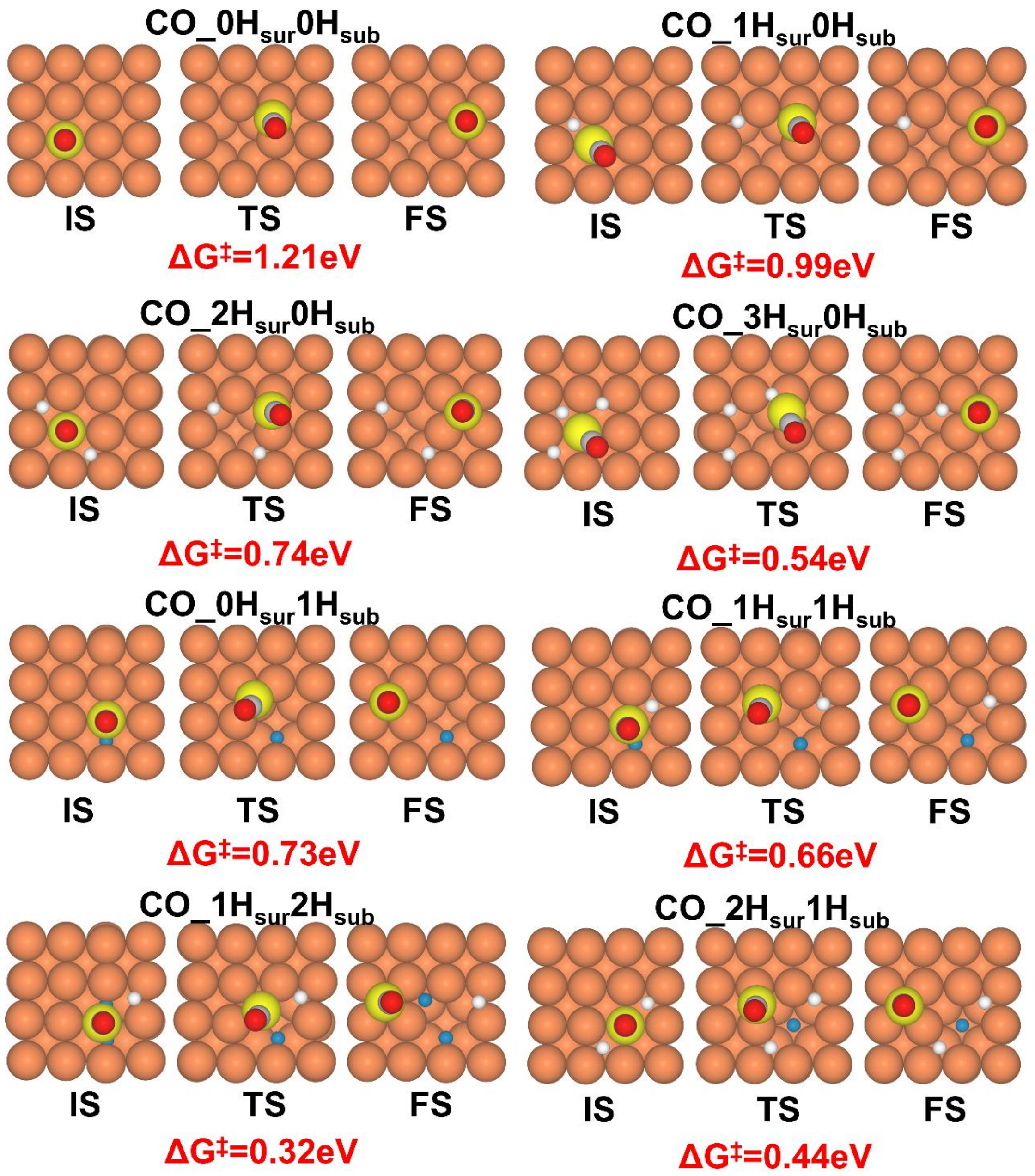

Figure S29. Atomic-scale pathways and energy barriers for Cu(100) Cu adatom formation in the presence of CO. Initial (IS), transition (TS), and final (FS) states for Cu adatom formation under varying H coverages. Color code: Cu (brown), the dissolving Cu atom (yellow), H_{sur}(white), H_{sub}(blue).

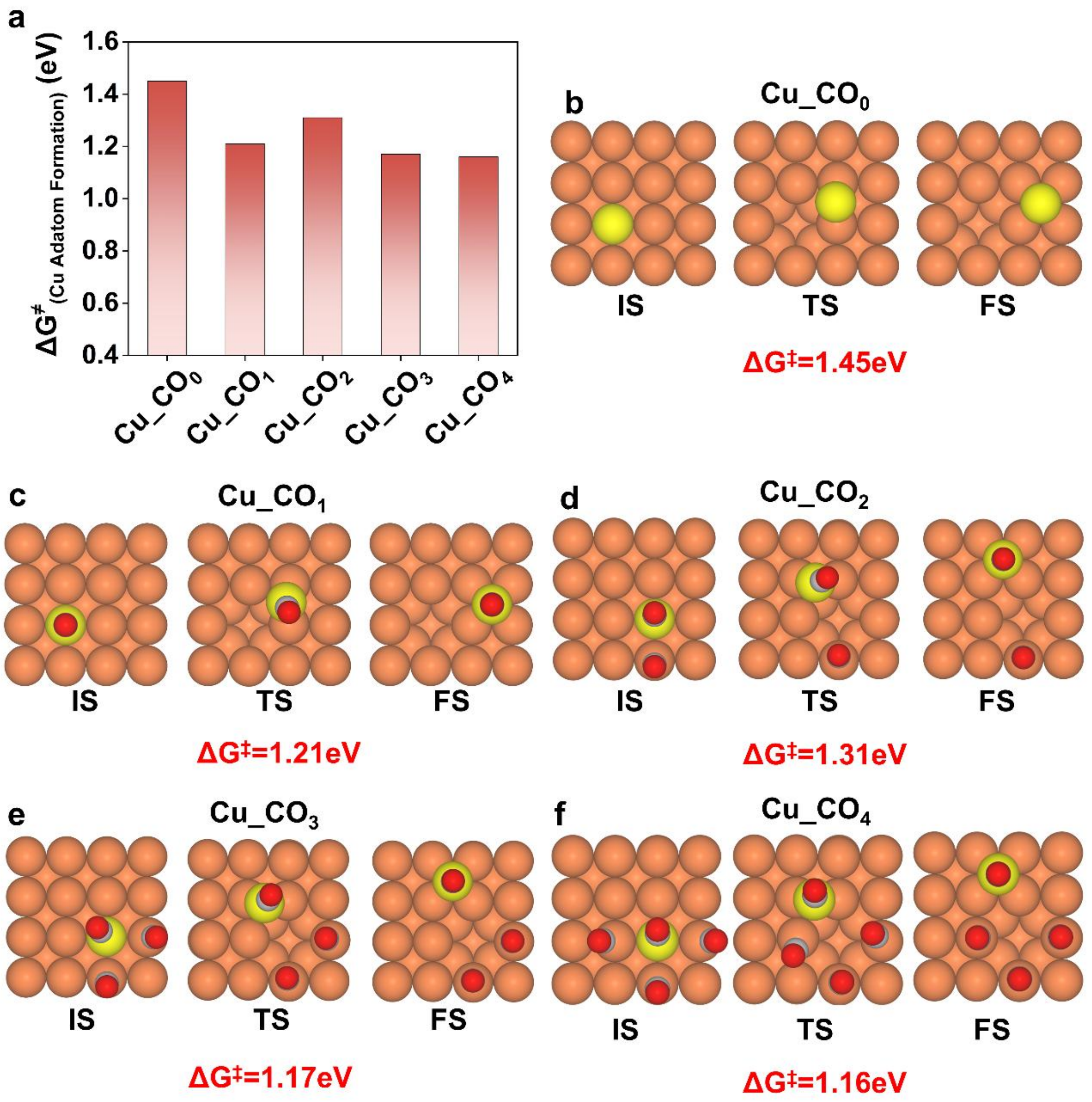

Figure S30. Energy barriers for Cu adatom formation under different CO coverages. (a) Summary of calculated energy barriers across varying CO coverage levels. (b–f) Schematics illustrating the Cu adatom formation process and corresponding energy profiles for each specific CO coverage condition. Color code: Cu (brown), the dissolving Cu atom (yellow), C (gray), O (red).

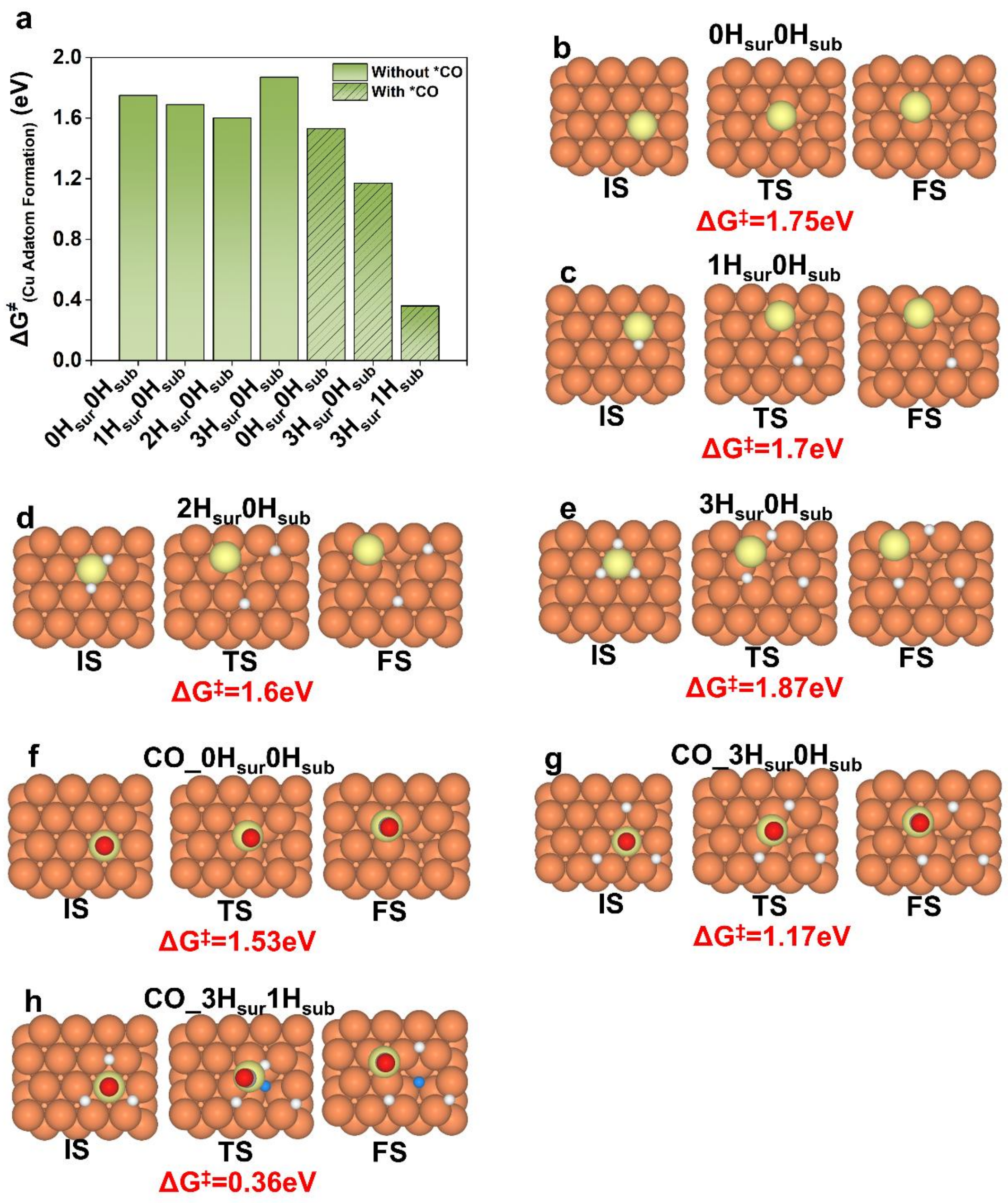

Figure S31. (a) Summary of the calculated energy barriers for Cu adatom formation on the Cu(111) surface under various co-adsorption environments involving *CO, $*H_{sur}$, and $*H_{sub}$. (b-h) Atomic configurations, energy diagrams, and corresponding quantitative barriers illustrating the initial state (IS), transition state (TS), and final state (FS) for each specific adsorption condition. Color code: Cu (brown), the dissolving Cu atom (yellow), H_{sur}(white), H_{sub}(blue).

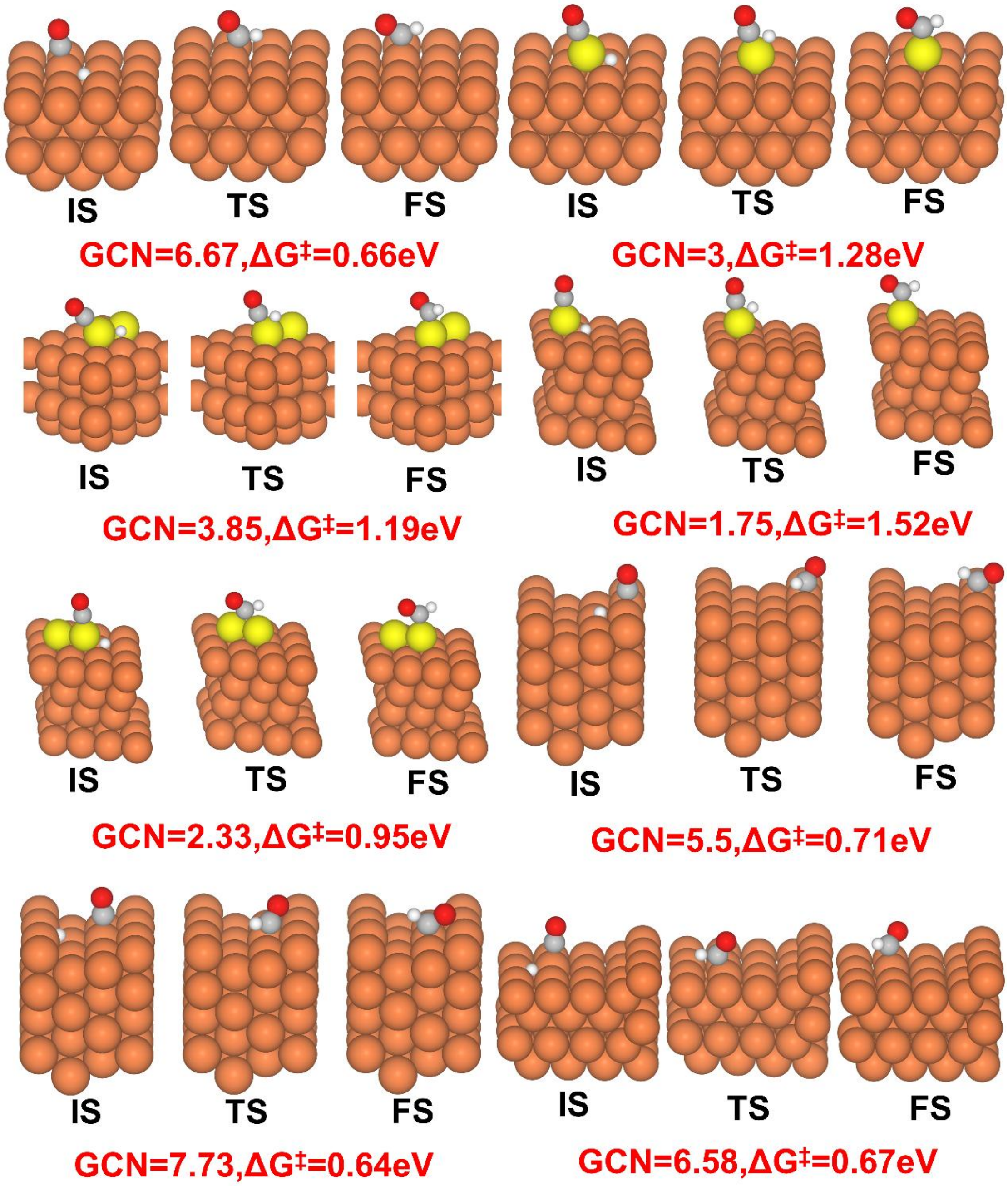

Figure S32. Generalized coordination number-dependent activity for *CO hydrogenation via LH mechanism. Reaction pathways of *CO conversion to *CHO across surfaces with varying generalized coordination numbers. Color code: Cu (brown), Cu adatom (yellow), C (gray), O (red), H (white).

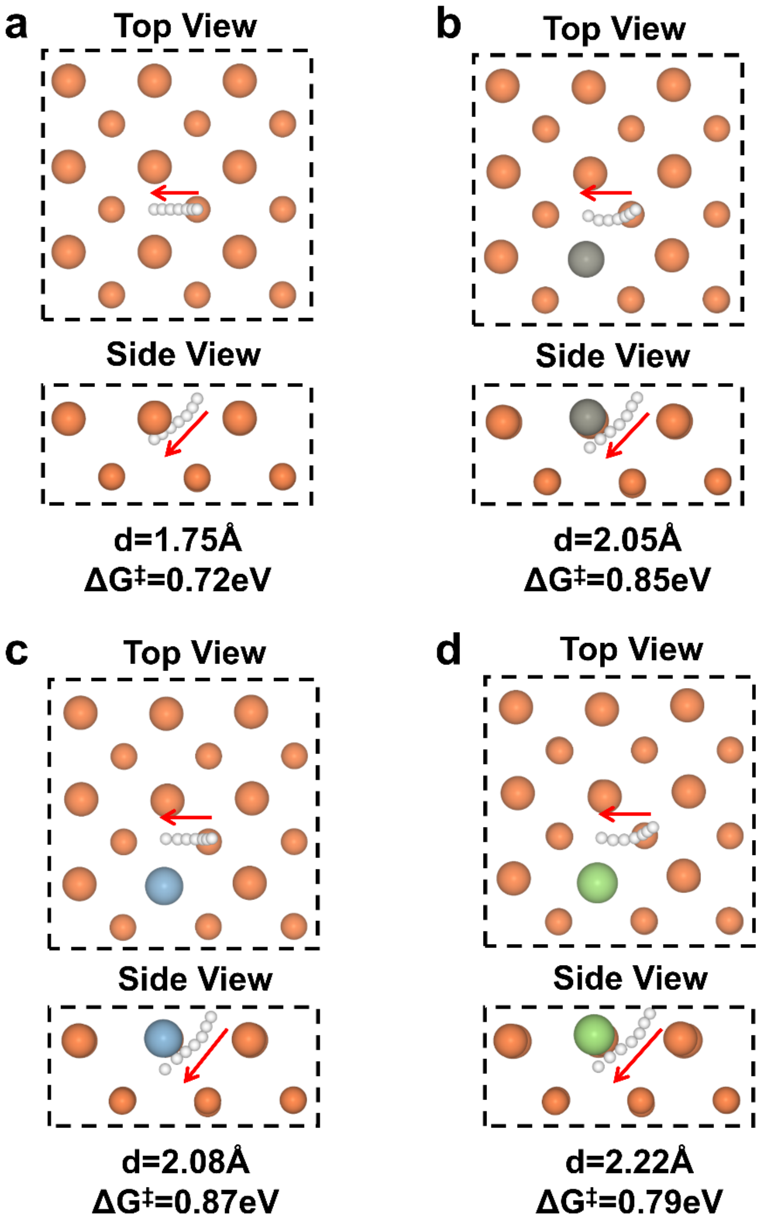

Figure S33. Alloying effects on hydrogen migration energetics in Cu-based bimetallic systems. Hydrogen migration pathways and corresponding energy barriers for (a) pure Cu and (b-d) Cu-based alloy surfaces, modeled by substituting a single surface Cu atom with Zn, Al, or Ga, respectively. Atomic configurations (top and side views) along the pathways are displayed. Color code: Cu (brown), Zn (gray), Al (blue), Ga (green), H (white).

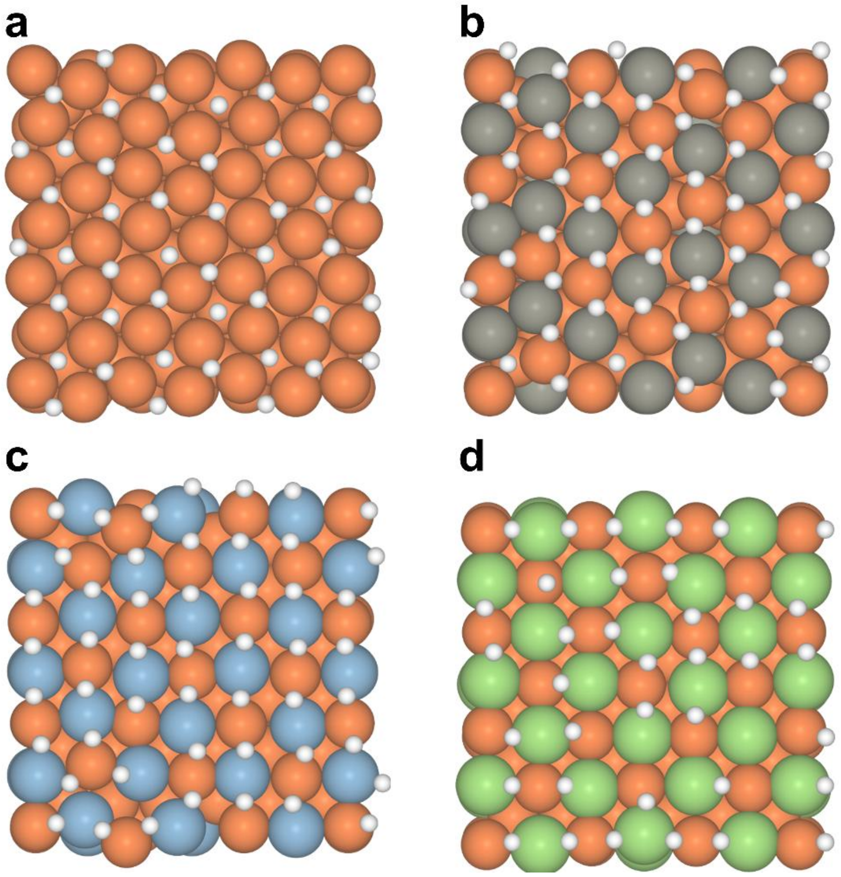

Figure S34. Hydrogen-induced surface reconstruction and evolution of the coordination environment in bimetallic catalysts. Atomic configurations after 10 ps AIMD simulations with 1 ML of H coverage on (a) 6×6 Cu(100), (b) 6×6 CuZn(100), (c) 6×6 CuAl(100), and (d) 6×6 CuGa(100) surfaces. All bimetallic surfaces possess a 1:1 Cu:M stoichiometric ratio. Color code: Cu (brown), Zn (gray), Al (blue), Ga (green), H (white).

Reference

1. Zhang Y, Wang H, Chen W *et al.* DP-GEN: A concurrent learning platform for the generation of reliable deep learning based potential energy models. *Comput Phys Commun* 2020; **253**: 107206.
2. Zheng S, Zhang X-M, Liu H-S *et al.* Active phase discovery in heterogeneous catalysis via topology-guided sampling and machine learning. *Nat Commun* 2025; **16**: 2542.
3. Weisfeiler B, Leman A. The reduction of a graph to canonical form and the algebra which appears therein. *nti, Series* 1968; **2**: 12-16.
4. Plimpton S. Fast parallel algorithms for short-range molecular dynamics. *J Comput Phys* 1995; **117**: 1-19.
5. Fu F, Wang X, Zhang L *et al.* Unraveling the atomic-scale mechanism of phase transformations and structural evolutions during (de)lithiation in Si anodes. *Adv Funct Mater* 2023; **33**: 2303936.
6. Hou P, Yu Q, Luo F *et al.* Reactant-induced dynamic active sites on Cu catalysts during the water–gas shift reaction. *ACS Catal* 2025; **15**: 352-360.
7. Kresse G, Furthmüller J. Efficiency of ab-initio total energy calculations for metals and semiconductors using a plane-wave basis set. *Comput Mater Sci* 1996; **6**: 15-50.
8. Blöchl PE. Projector augmented-wave method. *Phys Rev B* 1994; **50**: 17953-17979.
9. Kresse G, Joubert D. From ultrasoft pseudopotentials to the projector augmented-wave method. *Phys Rev B* 1999; **59**: 1758-1775.
10. Zhang Y, Yang W. Comment on "generalized gradient approximation made simple". *Phys Rev Lett* 1998; **80**: 890-890.
11. Monkhorst HJ, Pack JD. Special points for brillouin-zone integrations. *Phys Rev B* 1976; **13**: 5188-5192.
12. Grimme S, Antony J, Ehrlich S *et al.* A consistent and accurate ab initio parametrization of density functional dispersion correction (dft-d) for the 94 elements h-pu. *J Chem Phys* 2010; **132**: 154104.
13. Wang H, Zhang L, Han J *et al.* Deepmd-kit: A deep learning package for many-body potential energy representation and molecular dynamics. *Comput Phys Commun* 2018; **228**: 178-184.
14. Lachet Vr, Boutin A, Tavitian B *et al.* Grand canonical monte carlo simulations of adsorption of mixtures of xylene molecules in faujasite zeolites. *Faraday Discuss* 1997; **106**: 307-323.
15. Xu J, Xie W, Han Y *et al.* Atomistic insights into the oxidation of flat and stepped platinum surfaces using large-scale machine learning potential-based grand-canonical monte carlo. *ACS Catal* 2022; **12**: 14812-14824.
16. Gai L, Shin YK, Raju M *et al.* Atomistic adsorption of oxygen and hydrogen on platinum catalysts by hybrid grand canonical monte carlo/reactive molecular dynamics. *J Phys Chem C* 2016; **120**: 9780-9793.
17. Fantauzzi D, Krick Calderón S, Mueller JE *et al.* Growth of stable surface oxides on Pt(111) at near-ambient pressures. *Angew Chem Int Ed* 2017; **56**: 2594-2598.
18. Steinmann SN, Michel C, Schwiedernoch R *et al.* Impacts of electrode potentials and solvents on the electroreduction of CO_2: A comparison of theoretical approaches. *Phys Chem*

Chem Phys 2015; **17**: 13949-13963.
19. Mathew K, Kolluru VSC, Mula S *et al*. Implicit self-consistent electrolyte model in plane-wave density-functional theory. *J Chem Phys* 2019; **151**: 234101.
